# Integrated Phytochemical Profiling, In Vitro Bioactivity, Molecular Docking, and Pharmacokinetic–Toxicity Evaluation of *Ficus carica* Leaf Extracts

**DOI:** 10.3390/life16091518

**Published:** 2026-09-11

**Authors:** Bihter Şahin, Zafer Maşlakcı, Gülşah Kani, Özge Tokul Ölmez

**Affiliations:** 1 Department of Chemistry and Chemical Processing Technologies, Susurluk Agriculture and Forest Vocational School, Bandırma Onyedi Eylül University, 10600 Balıkesir, Türkiye; zafer.maslakci@balikesir.edu.tr (Z.M.); gulsahkani954@gmail.com (G.K.); 2 Department of Chemistry and Chemical Processing Technologies, Susurluk Agriculture and Forest Vocational School, Balıkesir University, 10602 Balıkesir, Türkiye; 3 Department of Chemistry, Faculty of Science, Muğla Sıtkı Koçman University, 48121 Muğla, Türkiye; ozgetokul@mu.edu.tr

**Keywords:** *Ficus carica*, HPLC-DAD, antioxidant activity, enzyme inhibition activity, molecular docking, chemometric approach

## Abstract

*Ficus carica* L., a widely cultivated member of the *Moraceae* family, is recognized for its rich phytochemical composition and diverse biological activities; however, the influence of harvest season and extraction solvent on its bioactive profile remains insufficiently explored. In this context, leaves collected from Dalaman (Muğla, Türkiye) in April and November were comparatively evaluated in terms of phenolic profiles, antioxidant capacity, enzyme inhibitory potential, molecular docking interactions, and preliminary ADMET properties. Eight extracts were prepared using ethyl acetate, methanol, aqueous ethanol, and water, and phenolic composition was characterized by HPLC-DAD using 42 reference standards. Major contributors to bioactivity included hesperidin, rutin, chlorogenic acid, cynarin, naringenin, and 4-hydroxyresorcinol. Antioxidant activity was assessed via DPPH˙, ABTS˙^+^, CUPRAC, and *β*-carotene–linoleic acid assays, while enzyme inhibitory activities against acetylcholinesterase, butyrylcholinesterase, and tyrosinase were determined. Hesperidin reached its highest concentration in the water extract FC14 (80.06 mg/g). FC14 showed the strongest DPPH˙ (IC_50_: 54.46 ± 0.94 μg/mL), ABTS˙^+^ (IC_50_: 10.75 ± 0.17 μg/mL), and CUPRAC (A_0.5_: 95.38 ± 1.69 μg/mL) activities, whereas the ethyl acetate extract FC11 showed the strongest *β*-carotene–linoleic acid activity (IC_50_: 37.77 ± 2.11 μg/mL). The water extract FC24 showed the strongest AChE (IC_50_: 135.52 ± 0.77 μg/mL) and tyrosinase (IC_50_: 34.46 ± 1.05 μg/mL) inhibition. The results demonstrated that both harvesting season and extraction solvent significantly influenced the phytochemical composition and biological activities. Principal component analysis revealed clear discrimination among extracts, and molecular docking supported these findings by highlighting strong interactions of hesperidin and rutin with target enzymes. PCA explained 50.30% of the total variance. Molecular docking analysis revealed the strongest binding affinities for hesperidin toward AChE (−9.3 kcal/mol), hesperidin and rutin toward BChE (both −10.8 kcal/mol), and rutin toward tyrosinase (−10.3 kcal/mol). ADMET predictions suggested favorable oral drug-likeness for selected compounds, whereas others may require further optimization. Overall, these findings indicate that *F. carica* leaves represent a promising source of natural phenolics with multifunctional bioactivity.

## 1. Introduction

*Ficus carica* L. represents a key species within the genus *Ficus*. It is typically a deciduous plant and is widely known by its common name, “fig” [1]. One of the oldest fruit trees, the fig tree (*F. carica* L.) is prized for both its culinary and therapeutic qualities [2]. Comprising over 800 species of trees, shrubs, hemiepiphytes, climbers, and creepers distributed across tropical and subtropical regions, *Ficus* represents one of the most expansive genera among flowering plants [3]. *F. carica*, commonly known as the fig, is a deciduous species belonging to the Moraceae family and is consumed both fresh and dried. It is considered one of the earliest cultivated tree species in the world [4,5,6]. Renowned for its nutritional value, *F. carica* provides substantial amounts of vitamins, carbohydrates, minerals, sugars, phenolic constituents, organic acids, and lipids. Its fresh and dried fruits are particularly valued for their nutrient-rich composition [7,8,9].

Numerous pharmacological characteristics of *F. carica* have been reported. Numerous parts of this plant, including its leaves and roots, are used to treat inflammation, cardiovascular conditions, respiratory system disorders (such as sore throat, cough, and bronchial issues), gastrointestinal disorders (such as colic, indigestion, loss of appetite, and diarrhea), and as an antispasmodic. Antioxidant, antibacterial, neurodegenerative preventative, anti-arthritic, anti-cancer, anti-diabetic, and numerous other biological qualities have been discovered through research utilizing both in vitro and in vivo models [10,11,12,13,14,15].

*F. carica* contributes to cell regeneration, is important for the body’s daily vitamin needs, plays a role in balancing blood pressure and is known to be an effective food against cancer. It is grown in many provinces in our country. Phytochemical studies on the fruits and leaves of *F. carica* have revealed that the *F. carica* plant is rich in phenolics, organic acids and volatile compounds [16,17,18].

*F. carica* has been reported to alleviate a wide range of ailments, including endocrine disorders such as diabetes; gastrointestinal issues like ulcers and vomiting; respiratory conditions such as liver-related complications, asthma, and cough; and cardiovascular and reproductive problems, including menstrual pain. Additionally, it has been used to address various infectious diseases, including skin conditions, scabies, and gonorrhea. Phytochemical investigations of *F. carica* consistently demonstrate the presence of ceramides, cerebrosides, steroids, pentacyclic triterpenes, flavonoids, and various phenolic constituents [19,20,21,22]. Phytochemical investigations of *F. carica* have identified a diverse array of bioactive constituents, including phenolics, phytosterols, organic acids, anthocyanins, triterpenoids, coumarins, and various volatile compounds such as hydrocarbons and aliphatic alcohols, along with several additional classes of secondary metabolites distributed across different plant parts [23,24].

Li et al. (2021) [25] highlighted that *F. carica* leaves are traditionally consumed as herbal tea and employed in various medicinal applications. Their study indicated that the leaves confer therapeutic benefits in numerous conditions, including gastrointestinal, respiratory, cardiovascular, metabolic, and dermatological disorders, as well as ulcers, dysentery, hemorrhoids, cough, pulmonary diseases, and trauma-induced blood stasis. Moreover, the authors identified 126 chemical constituents in *F. carica* leaves and documented a wide spectrum of biological activities, such as antioxidant, anticancer, antidiabetic, hepatoprotective, anticholinesterase, anti-HSV-1, antibacterial, anti-inflammatory, and renoprotective effects. Nonetheless, they underscored the necessity for further studies to clarify the functional significance of these bioactive components [25].

The peel, pulp, leaves, and fruits of *F. carica* are known to contain a wide range of bioactive constituents, including cyanogenic compounds, chlorogenic acid, rutin, luteolin, and (+)-catechin, among others [26,27,28]. Fig-derived products have been traditionally used to manage various ailments, particularly dermatological conditions [29]. Extracts of *F. carica* have also demonstrated efficacy in alleviating symptoms of atopic dermatitis, suggesting their potential as alternatives to corticosteroid-based treatments [30]. Additionally, Al-Ogaili and colleagues reported that *F. carica* leaf extracts may contribute to the management of dietary and lifestyle-related disorders [31].

The juice obtained from *F. carica* leaves is used in the treatment of vitiligo, mainly due to the furanocoumarins, psoralen and daidzein contained in them [32,33]. The antioxidant capacity of the leaf extract was evaluated and reported in terms of vitamin C equivalents per gram of extract (mg/g) [34].

Review of the literature indicates that various phenolic acids including 3-*O*- and 5-*O*-caffeoylquinic acid, ferulic acid, quercetin-3-*O*-glucoside, quercetin-3-*O*-rutinoside, psoralen, and bergapten as well as several organic acids (oxalic, citric, malic, quinic, shikimic, and fumaric acids) have been isolated from *F. carica* leaf extracts [23]. Additionally, triterpenoids such as bauerenol, lupeol acetate, methyl maslinate, and oleanolic acid identified in *F. carica* leaves were reported to exhibit irritant effects in mouse ear models [35]. Although biological activity and target-binding affinity are important indicators of the potential of plant-derived bioactive compounds, these parameters alone are insufficient to assess their suitability for further pharmacological consideration. Pharmacokinetic behavior and safety characteristics may substantially influence the biological relevance and potential applicability of such compounds. Therefore, preliminary in silico ADMET evaluation, including absorption, distribution, metabolism, drug-likeness, and toxicity predictions, can complement biological and molecular docking findings by identifying potential pharmacokinetic limitations and safety-related alerts at an early stage. In this context, assessment of the major phenolic constituents of *F. carica* leaves was considered important to provide a more comprehensive evaluation of their potential biological and pharmaceutical relevance. In this study, we aimed to provide a comprehensive evaluation of the phytochemical composition and biological potential of *F. carica* leaves collected at different stages of the fruiting cycle. Particular emphasis was placed on characterizing their phenolic constituents and investigating their antioxidant, anticholinesterase, and tyrosinase inhibitory activities, together with chemometric analysis, molecular docking, and preliminary in silico ADMET assessment of selected major phenolic compounds. The ADMET analysis was incorporated to complement the biological and docking findings by evaluating predicted pharmacokinetic behavior, oral drug-likeness, and potential toxicity-related alerts.

## 2. Materials and Methods

### 2.1. Collection of F. carica Leaves and Preparation of Study Materials

*F. carica* leaves were collected from Dalaman district of Muğla, Türkiye, in April and November [dates: 15 April 2022 and 18 November 2022]. In both sampling periods, fully developed and healthy leaves at a similar phenological stage were selected to minimize variability due to leaf age. The leaves were collected from a single tree growing under similar environmental conditions. For each sampling period, leaves were gathered from different parts of the tree and pooled to obtain a sample representative of the population. The leaves were dried in the shade and at room temperature and made ready for analysis. Then, 30 g of plant material was used to obtain a total of 8 extracts from the dried *F. carica* leaves from April and November. *F. carica* was taxonomically identified based on morphological characteristics using standard botanical references. The extraction yields in 30 g of dried plant material for the ethyl acetate extracts were 2.84 ± 0.16% and 2.23 ± 0.17% for the April and November samples, respectively. For the methanol extracts, the yields were 6.12 ± 0.18% and 6.56 ± 0.21%, respectively, while the aqueous ethanol extracts yielded 7.26 ± 0.57% and 7.14 ± 0.61%, and the water extracts yielded 7.48 ± 0.73% and 7.16 ± 0.81% for the April and November samples, respectively. Although a voucher specimen was not deposited in a herbarium, the species is well-known and widely cultivated.

### 2.2. Extraction Process

*F. carica* leaves were subjected to sequential extraction using hexane, ethyl acetate, methanol, aqueous ethanol, and water. Prior to the extraction of the target compounds, the dried leaf material was treated with hexane to remove lipophilic components. Following hexane treatment, the plant material was sequentially extracted with ethyl acetate, methanol, aqueous ethanol, and water. For each extraction step, a solvent-to-plant material ratio of 5:1 (*v*/*w*) was used. The extraction process was repeated three times with fresh solvent until the color of the extract became markedly lighter (min × 3 times). The resulting extracts were combined separately according to the solvent used, and the solvents were removed under reduced pressure using a rotary evaporator. The obtained extracts were stored in a refrigerator until further analysis. All extractions were performed in 2025, with three independent extraction replicates prepared from separate portions of plant material. In the naming of the extracts, the first digit is 1 (April) or 2 (November), indicating the months in which the leaves were collected. The second digit indicates the type of solvent (1: ethyl acetate, 2: methanol, 3: aqueous ethanol, 4: water).

### 2.3. Preparation of Samples for HPLC Analysis

Firstly, 8 mg of extract was prepared by dissolving it in 1 mL of methanol. Ethyl acetate, methanol, aqueous ethanol and water extracts of *F. carica* leaves were homogenized in an ultrasonic bath at room-temperature conditions for 5 min, filtered through 0.45 μm PTFE filters and made ready for injection.

### 2.4. HPLC Analyses

Phenolic constituents of the ethyl acetate, methanol, aqueous ethanol, and water ex-tracts of *F. carica* leaves were analyzed using an HPLC-DAD system (Shimadzu Promi-nence-*i* LC-2030C 3D Plus, Shimadzu Corporation, Kyoto, Japan), following a method modified from Ozge Tokul-Olmez et al. [36]. Chromatographic separation was performed at 35 °C on an Inertsil ODS-3 C_18_ reversed-phase column (5 μm, 4.6 × 250 mm) equipped with an Inertsil C18 guard column. The mobile phase consisted of 0.1% acetic acid in water (A) and 100% methanol (B). A multistep gradient elution was applied at a flow rate of 1.0 mL/min with the following program: 0–6 min, 2% B; 7–11 min, 5% B; 12 min, 6% B; 13–24 min, 10% B; 25–31 min, 25% B; 32–36 min, 30% B; 37–42 min, 40% B; 43–47 min, 42% B; 48–52 min, 54% B; 53–62 min, 55% B; 63–66 min, 56% B; 67–71 min, 65% B; 72–74 min, 100% B; 75–76 min, 50% B; and finally returning to the initial conditions of 2% B at 77 min for column re-equilibration. The column temperature was maintained at 35 °C. The injection volume was set at 20 μL, and detection was carried out using a diode array detector (DAD) at 254 nm. The phenolic compounds were identified by comparing their retention times and UV spectra with those of 42 reference standards. A detailed description of the column program and validation parameters is provided in Tables S1 and S2.

### 2.5. Antioxidant and Enzyme Inhibitory Activity

The detailed procedures for all experiments are provided in the Supplementary Materials [37,38,39,40,41,42].

### 2.6. Statistical Analysis

Data analyses were presented as mean ± standard deviation (SD) based on three independent replicates. All statistical analyses, including normality testing, variance analysis, correlation, and multivariate modeling, were performed using Minitab Software version 17.1.0 (Minitab Inc., State College, PA, USA, 2013). To determine statistically significant differences in both bioactive compound concentrations and biological activities among the eight distinct extracts, one-way analysis of variance (ANOVA) was performed, followed by Tukey’s post-hoc multiple comparison test with the significance threshold set at *p* < 0.001. Prior to the correlation analysis, the normality of the data distribution was verified using the Ryan-Joiner test, which serves as a powerful alternative similar to the Shapiro–Wilk test. Subsequently, Pearson’s correlation analysis was conducted to evaluate the relationships between individual bioactive compounds and biological (antioxidant and enzyme inhibition) activities, with the statistical significance threshold strictly set at *p* < 0.05 across all analyses. In addition, Principal Component Analysis (PCA) was applied to thoroughly evaluate the multivariate relationships between phenolic contents, antioxidant activities, and enzyme inhibition results, as well as to reveal similarities and differences among the eight extracts. Before PCA, the dataset was standardized, and the analysis was performed based on the correlation matrix.

### 2.7. Molecular Docking Simulations

Molecular docking is a computational approach extensively employed in drug development and structural biology to estimate the binding pose and interaction strength between a small molecule (ligand) and a target macromolecule (receptor), most commonly a protein. This strategy plays a crucial role in structure-based drug design, optimization of lead compounds, and virtual screening workflows [43,44]. The three-dimensional crystallographic structures of the selected enzymes were retrieved from the Protein Data Bank (https://www.rcsb.org/, accessed on 15 August 2026) using their corresponding PDB accession numbers: 6O52 for AChE, 4BDS for BChE, and 2Y9X for tyrosinase. Crystallographic waters and ions were deleted, and hydrogen atoms were introduced at a neutral pH of 7.0. The docking grid was defined based on the active-site coordinates of the ligands in the crystal structures of the enzymes. A set of 10 poses was generated for each ligand, and the pose with the lowest ΔG binding score was selected for detailed evaluation. This workflow helped identify the most energetically favorable docking configurations and highlighted the key interaction features between the ligands and the protein. The three-dimensional structures of the compounds were also generated, followed by hydrogen addition and geometry optimization using Discovery Studio Visualizer 2020 software [45]. Subsequently, molecular docking simulations were conducted with AutoDock 4.2. [46] under standard experimental protocols.

AutoDock uses an empirical free-energy scoring approach to estimate the binding affinity between ligands and receptors. This model incorporates van der Waals and electrostatic interactions, hydrogen bonding, desolvation terms, and torsional entropy penalties. The total binding free energy (ΔG_binding) is derived from the cumulative contribution of these terms, providing an approximate prediction of binding affinity and the inhibition constant (Ki) for each ligand. Through this empirical scoring function, AutoDock can evaluate docking poses and approximate the inhibition constant (Ki), thereby providing an estimate of ligand binding affinity based on thermodynamic principles. Using this scoring framework, AutoDock ranks the poses and calculates a Ki value for each ligand.

To predict the pharmacokinetic, drug-likeness, and toxicological properties of the selected bioactive phenolic compounds identified in *F. carica* leaf extracts, in silico ADMET analyses were performed using the SwissADME web tool (http://www.swissadme.ch/, accessed on 15 August 2026), pkCSM (https://biosig.lab.uq.edu.au/pkcsm/, accessed on 15 August 2026) and ProTox-II (https://tox-new.charite.de/protox_II/, accessed on 15 August 2026) online platforms. The selected compounds included hesperidin, rutin, chlorogenic acid, cynarin, ellagic acid, kaempferol, naringenin, and ferulic acid. SwissADME was used to estimate key physicochemical and drug-likeness parameters, including molecular weight, hydrogen bond acceptors and donors, topological polar surface area, number of rotatable bonds, consensus LogP, water solubility, gastrointestinal absorption, blood–brain barrier permeability, P-glycoprotein substrate status, Lipinski’s rule of five violations, and bioavailability score. In addition, pharmacokinetic-related properties such as intestinal absorption, distribution volume, CYP enzyme inhibition, total clearance, and toxicity-related parameters were evaluated. The ProTox-II web server was further employed to predict the toxicological profiles of the selected compounds, including predicted LD_50_ values, toxicity class, hepatotoxicity, carcinogenicity, immunotoxicity, mutagenicity, and cytotoxicity. These computational predictions provided supportive information regarding the oral bioavailability, safety profile, and drug-likeness potential of the major phenolic constituents of *F. carica* leaf extracts.

## 3. Results and Discussion

### 3.1. Phenolic Composition of F. carica Leaf Extracts by HPLC-DAD

*F. carica* leaves collected in April (FC11, FC12, FC13, FC14) and November (FC21, FC22, FC23, FC24) were first subjected to sequential maceration, during which the lipophilic components were removed using hexane. The phenolic profiles of all extracts were analyzed, and the results are presented in Table 1. The HPLC-DAD data were quantified in mg/g, and compounds marked as “*tr*” indicate trace-level detection. All chromatograms were recorded at 254 nm, and the representative chromatograms are shown in Figures S1 and S2.

Cynarin, known for its strong antioxidant activity, was identified as the major compound in the FC11 extract with a concentration of 5.89 mg/g. It was followed by naringenin (2.44 mg/g), protocatechuic acid (2.44 mg/g), and chlorogenic acid (0.31 mg/g). Oleuropein and curcumin were not detected in FC11, while vanillic acid, vanillin, and ferulic acid were present in trace amounts. In the FC21 extract, 4-hydroxyresorcinol (18.94 mg/g) was the predominant phenolic compound, followed by curcumin (2.38 mg/g), oleuropein (0.59 mg/g), naringenin (0.97 mg/g), and chlorogenic acid (0.28 mg/g). Among the two extracts, naringenin and chlorogenic acid were detected in both, representing common phenolic constituents.

FC12 methanol extract, chlorogenic acid was the predominant compound (8.99 mg/g), followed by cynarin (5.89 mg/g), rutin (2.91 mg/g), ferulic acid (1.90 mg/g), kaempferol (1.53 mg/g), and 4-hydroxyresorcinol (1.47 mg/g). In contrast, 4-hydroxyresorcinol (25.17 mg/g) was the major phenolic compound in the FC22 extract, followed by cynarin (6.23 mg/g), rutin (2.45 mg/g), curcumin (2.59 mg/g), and epicatechin (1.72 mg/g).

Both extracts shared several common phenolic constituents, including protocatechuic acid, 4-hydroxybenzaldehyde, chlorogenic acid, ferulic acid, cynarin, rutin, and 4-hydroxyresorcinol. Overall, the FC12 extract exhibited a richer phenolic profile compared to FC22. The corresponding chromatograms are presented in Figures S3 and S4. In FC13 aqueous ethanol extract, hesperidin was identified as the major compound (39.52 mg/g), followed by rutin (2.15 mg/g), fumaric acid (1.74 mg/g), and luteolin (0.89 mg/g). In the FC23 extract, cynarin (6.33 mg/g) was the predominant compound, followed by 4-hydroxyresorcinol (5.71 mg/g), naringenin (4.46 mg/g), curcumin (0.96 mg/g), and chlorogenic acid (0.91 mg/g). Chlorogenic acid, rutin, and protocatechuic acid were detected as common constituents in both aqueous ethanol extracts. The corresponding chromatograms are presented in Figures S5 and S6.

In the FC14 water extract, hesperidin was determined as the major compound (80.06 mg/g), followed by cynarin (5.95 mg/g), ellagic acid (3.72 mg/g), rutin (1.23 mg/g), epicatechin (0.56 mg/g), and chlorogenic acid (0.50 mg/g). In the FC24 extract, hesperidin remained the predominant compound (42.17 mg/g), followed by rutin (0.69 mg/g), 4-hydroxyresorcinol (0.36 mg/g), and cynarin. Common phenolic constituents identified in both extracts included hesperidin, rutin, protocatechuic acid, chlorogenic acid, and fisetin. Overall, the FC24 extract exhibited the lowest phenolic content, which is reflected in its activity results. The corresponding chromatograms are presented in Figures S7 and S8.

Hesperidin exhibits notable antioxidant and anti-inflammatory activities, which underpin its diverse biological effects in experimental models of cardiovascular disorders [47,48] and diabetes [49,50], as well as its potential role in cancer prevention [48]. Moreover, these antioxidant and anti-inflammatory mechanisms contribute to the improvement of symptoms in animal models of neurodegenerative conditions, including Alzheimer’s disease [51]. Hesperidin was also identified as one of the major allelochemicals associated with *F. carica* roots. Li et al. (2024) [52] reported hesperidin, together with bergapten, psoralen, and umbelliferone, among the principal compounds released from *F. carica* roots. In the present study, hesperidin was detected at a particularly high level of 80.06 mg/g in the FC14 extract, highlighting the considerable accumulation of this flavonoid in *F. carica* leaves. Although the plant part and analytical approaches differed between the studies, the identification of hesperidin in both root-associated and leaf matrices suggests that this compound may represent an important secondary metabolite of *F. carica* [52]. Rutin is a well-recognized antioxidant belonging to the flavonoid group; however, its application in cosmetic dermal formulations is limited by its poor aqueous solubility [53]. Flavonoids, particularly rutin, have demonstrated pronounced antioxidant properties at the molecular level in various studies [54]. The remarkable antioxidant activity of rutin is attributed to its phenolic structure, characterized by an aromatic ring bearing a hydroxyl group capable of donating electrons and hydrogen atoms [55]. However, the limited skin permeation capacity of rutin restricts its efficient use in dermal formulations [56]. Rutin was also reported as an important phenolic constituent of *F. carica* leaves by Yu et al. (2020) [57], who obtained a rutin extraction yield of 4.77 mg/g using an optimized PEG8000-based surfactant-assisted microwave extraction method. This finding is consistent with the detection of rutin in the present study and further supports its occurrence as a characteristic flavonoid in *F. carica* leaves [57]. The differences in rutin levels between the two studies may be associated with variations in extraction solvent, extraction technique, harvesting period, and geographical origin of the plant material.

The varying phytochemical profiles reveal that the exceptional biological and antioxidant traits of certain fractions, such as FC23 (November, aqueous ethanol), do not rely on common reference compounds like hesperidin and fisetin.

Under the rules of sequential extraction, phytochemical partitioning is highly sensitive to seasonal matrix conditions. In the April harvest, hesperidin was heavily extracted in the intermediate polar phases (FC13: 39.52 mg/g; FC14: 80.06 mg/g). In contrast, during November, hesperidin bypassed the aqueous ethanol extract (FC23) entirely and locked selectively into the absolute water extract (FC24: 42.17 mg/g).

This structural divergence suggests that the biological activities of the extracts may be associated with their distinct chemical profiles. For example, FC23, which did not contain hesperidin, showed considerable enzyme inhibitory and radical scavenging activities and was characterized by relatively high levels of other phenolic compounds. In particular, FC23 contained the highest concentrations of cynarin (6.33 mg/g) and naringenin (4.46 mg/g) among the November extracts, together with chlorogenic acid (0.91 mg/g). These findings suggest that the observed biological activities may be related to the combined presence of several phytochemicals rather than to a single compound. However, the present results indicate associations between phytochemical composition and biological activity and do not establish direct synergistic or mechanistic effects. To assess whether the observed differences in phytochemical composition were statistically significant, one-way ANOVA followed by Tukey’s post-hoc test was performed. The results showed that the harvest period and extraction sequence significantly affected the concentrations of the quantified phytochemicals (*p* < 0.05). These differences in phenolic composition provided the chemical basis for the subsequent evaluation of antioxidant and enzyme inhibitory activities.

### 3.2. Antioxidant and Enzyme Inhibitory Activities

*F. carica* leaves were collected in April and November, and a total of eight extracts with varying polarities were prepared using four different solvents. The antioxidant activities of all extracts were evaluated using four distinct assays: DPPH˙, ABTS˙^+^, *β*-carotene–linoleic acid, and CUPRAC. The corresponding IC_50_ values are presented in Table 2. According to the DPPH˙ assay, the FC13 (IC_50_: 57.54 ± 0.02 μg/mL) and FC14 (IC_50_: 54.46 ± 0.94 μg/mL) extracts exhibited the strongest antioxidant activity. In the ABTS˙^+^ assay, FC14 showed the highest activity (IC_50_: 10.75 ± 0.17 μg/mL), followed by FC23 (IC_50_: 59.88 ± 1.48 μg/mL), FC24 (IC_50_: 61.35 ± 0.27 μg/mL), FC13 (IC_50_: 63.50 ± 0.66 μg/mL), and FC22 (IC_50_: 96.75 ± 0.03 μg/mL). In the *β*-carotene–linoleic acid assay, FC21 showed no activity, whereas FC11 exhibited the highest activity (IC_50_: 37.77 ± 2.11 μg/mL), followed by FC24 (IC_50_: 48.39 ± 1.54 μg/mL), FC13 (IC_50_: 65.23 ± 0.76 μg/mL), FC23 (IC_50_: 73.53 ± 0.55 μg/mL), and FC22 (IC_50_: 75.53 ± 0.99 μg/mL). In the CUPRAC assay, the strongest antioxidant activity was observed in the FC14 extract (A_0.5_: 95.38 ± 1.69 μg/mL).

*F. carica* leaf extracts exhibited notable antioxidant activities, with methanol and aqueous ethanol extracts (particularly FC13 and FC14) showing the strongest effects in DPPH˙, ABTS˙^+^, *β*-carotene–linoleic acid, and CUPRAC assays. The phenolic profiling revealed that compounds such as cynarin, hesperidin, rutin, chlorogenic acid, and 4-hydroxyresorcinol were predominant and likely contributed to the observed bioactivities. These findings highlight the potential of *F. carica* leaves as a source of natural antioxidants and support their traditional medicinal use.

Acetylcholinesterase, butyrylcholinesterase and tyrosinase enzyme inhibition activities of 8 extracts obtained from *F. carica* were performed. All IC_50_ results obtained are given in Table 2. According to the acetylcholinesterase enzyme inhibition activity results of the 8 extracts obtained. It is seen that the water extract FC24 (IC_50_: 135.52 ± 0.77 μg/mL) collected in November gave the best activity. According to the results of butyrylcholinesterase enzyme inhibition activity, FC12 (IC_50_: 181.02 ± 0.34 μg/mL) methanol extract showed the best activity. Galantamine was used as the standard reference substance.

The activity results of tyrosinase enzyme inhibition of ethyl acetate, methanol, aqueous ethanol and water extracts of *F. carica* leaves collected in April showed that FC13 extract showed the best activity (IC_50_: 195.77 ± 2.09 µg/mL). In the activity results of tyrosinase enzyme inhibition of ethyl acetate, methanol, aqueous ethanol and water extracts of *F. carica* leaves collected in November, the best activity was, respectively, FC24 extract (IC_50_: 34.46 ± 1.05 µg/mL), FC22 extract (IC_50_: 38.17 ± 0.97 µg/mL) and FC23 extract (IC_50_: 88.57 ± 1.46 µg/mL). Kojic acid was used as a standard reference substance in the tyrosinase enzyme inhibition assay.

To further investigate whether the differences in biological activities were associated with variations in phenolic composition, Pearson correlation, PCA, and HCA were subsequently applied to the combined chemical and biological dataset.

### 3.3. Statistical Analysis of Chemical Composition and Biological Activities

#### 3.3.1. Pearson Correlation Analysis

**Figure 1:**
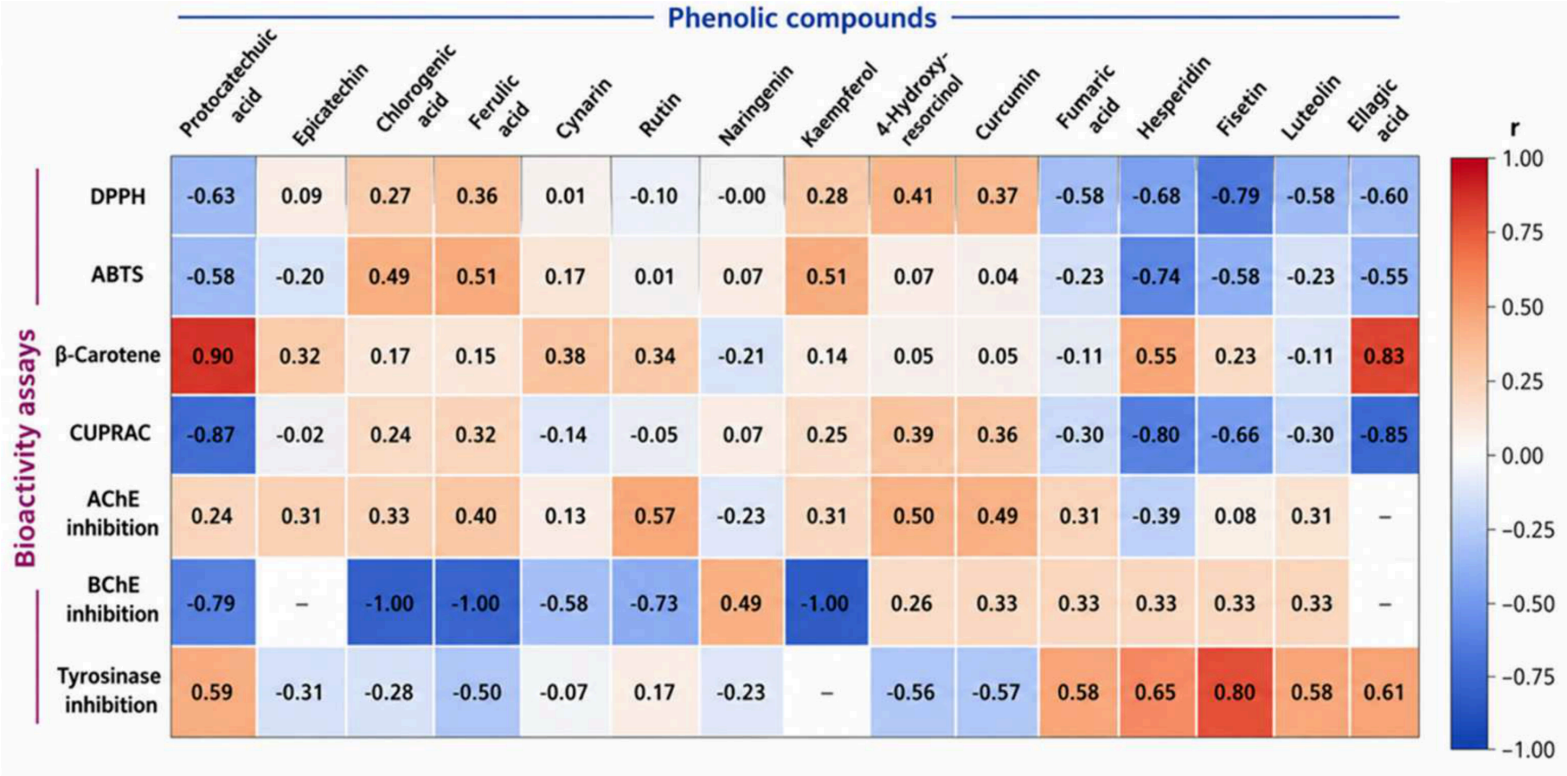
Pearson correlations between individual bioactive compounds, antioxidant and enzyme properties.

To understand the relationship between structure and activity, we performed Pearson’s correlation analysis. First, a potential correlation between individual bioactive compounds and antioxidant activities (DPPH˙, ABTS˙^+^, β-carotene–linoleic acid, and CUPRAC) as well as enzyme inhibitory activities (acetylcholinesterase, butyrylcholinesterase, and tyrosinase) was evaluated. For the mathematical analysis, the values indicated as “tr” (trace amounts) and “−” (not detected) in the datasets were treated as 0 (zero). The values reported as “>200” in the bioactivity results (indicating no or very weak activity) were numerically assigned as the upper limit value of 200. Data points labeled as “NA” (not analyzed) were excluded from the analysis, and calculations were performed only on the common samples with available data. In the heatmap, blue tones (negative correlations) indicate that as the concentration of the related phenolic compound increases, the IC_50_ value decreases, corresponding to an enhancement in biological activity. In contrast, red tones represent the opposite effect, indicating reduced activity or potential antagonistic interactions. Accordingly, the heat map we obtained as a result of Pearson correlation is given in Figure 1.

Based on the correlation analysis, several phenolic compounds exhibited relatively strong associations with the investigated biological activities. Hesperidin was negatively correlated with ABTS˙^+^ (r = −0.74) and CUPRAC (r = −0.80) values, suggesting a relationship between hesperidin concentration and these antioxidant-related measurements. Protocatechuic acid (r = −0.87) and ellagic acid (r = −0.85) likewise displayed strong negative correlations with CUPRAC values. These relationships reflect associations between the concentrations of these compounds and CUPRAC values rather than demonstrating direct contributions to antioxidant activity.

For BChE, chlorogenic acid, ferulic acid, and kaempferol exhibited very strong negative correlations with BChE-related IC_50_ values (r = −1.00 for each), while protocatechuic acid (r = −0.79) and rutin (r = −0.73) were also negatively associated with BChE-related IC_50_ values. These correlations may reflect the involvement of these compounds in the observed inhibitory activity. Fisetin, a polyphenolic flavonoid identified in the present study, has attracted considerable attention due to its antioxidant and neuroprotective properties. Vishwas et al. (2024) [58] demonstrated that fisetin nanoemulsion significantly attenuated oxidative stress, acetylcholinesterase (AChE), *β*-amyloid accumulation, and neuroinflammation in an AlCl_3_-induced Alzheimer’s disease rat model, accompanied by improvements in cognitive functions. These findings suggest that the presence of fisetin in *F. carica* leaves may contribute to the antioxidant and potential neuroprotective properties of the plant [58].

Fisetin was negatively correlated with DPPH˙ values (r = −0.79), indicating a relationship between fisetin concentration and DPPH˙ radical scavenging activity across the investigated extracts. However, this correlation does not imply that fisetin alone determines antioxidant activity, particularly because fisetin was not detected in some extracts, including F23. Overall, the observed antioxidant activity is likely related to the combined effects of multiple phenolic constituents and their potential interactions.

These multivariate analyses were performed to integrate the HPLC-DAD and bioactivity data and to identify overall patterns among the eight extracts.

#### 3.3.2. Principal Component Analysis (PCA)

PCA was applied to comprehensively evaluate the relationship between the phenolic compound contents and antioxidant and enzyme inhibitor activities of eight extracts (FC11, FC12, FC13, FC14, FC21, FC22, FC23, FC24) obtained from *F. carica* leaves harvested at different times and using different solvents. The analysis revealed that seven principal components with eigenvalues greater than one were retained (Table S3). The first principal component accounted for 30.0% of the total variance, while the first two components together explained 50.30% of the total variance. The fact that these two components together explained more than half of the dataset indicates that the resulting loading and scoring plots reliably reflect the chemical and biological differences between the extracts.

**Figure 2:**
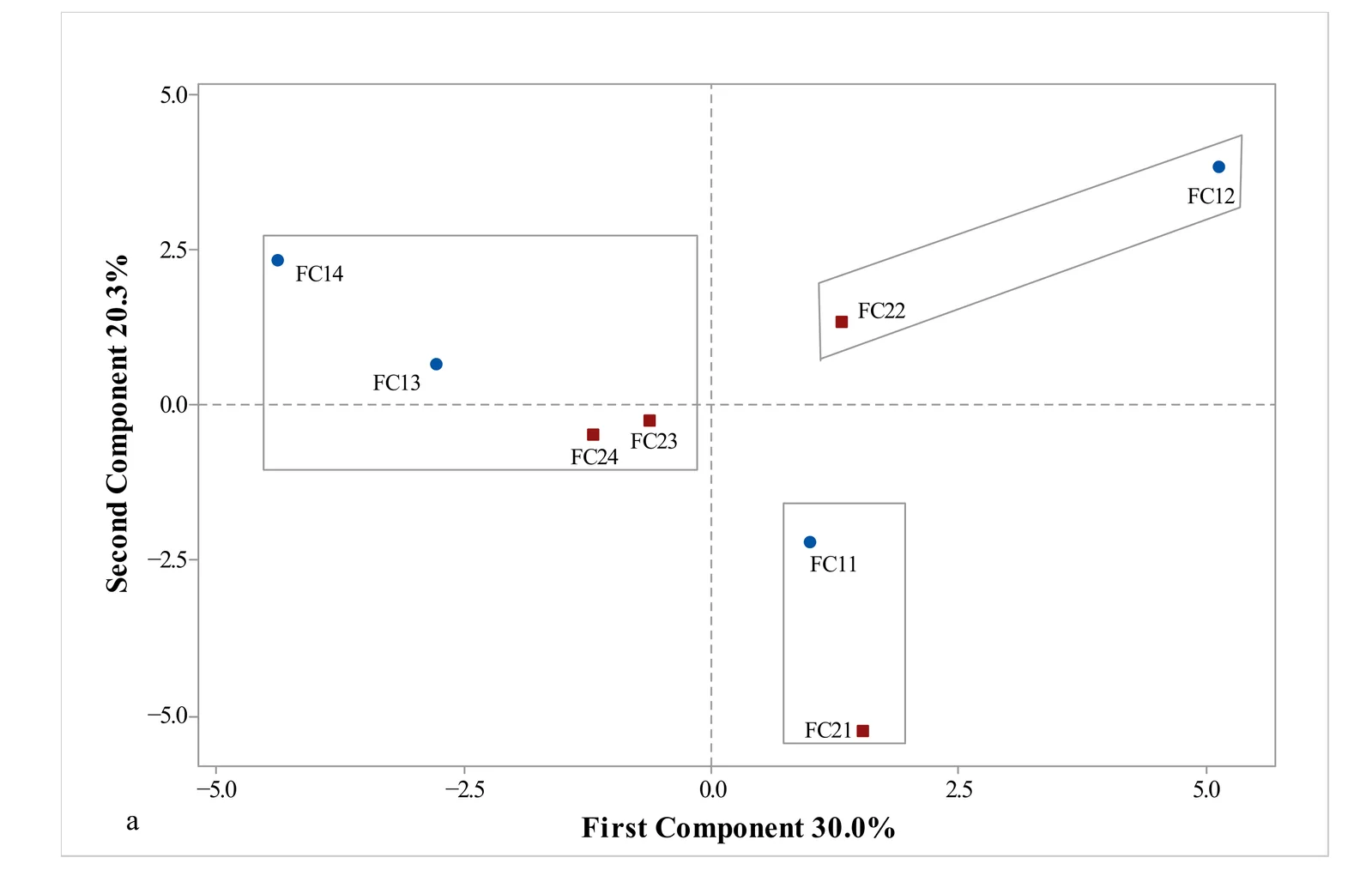

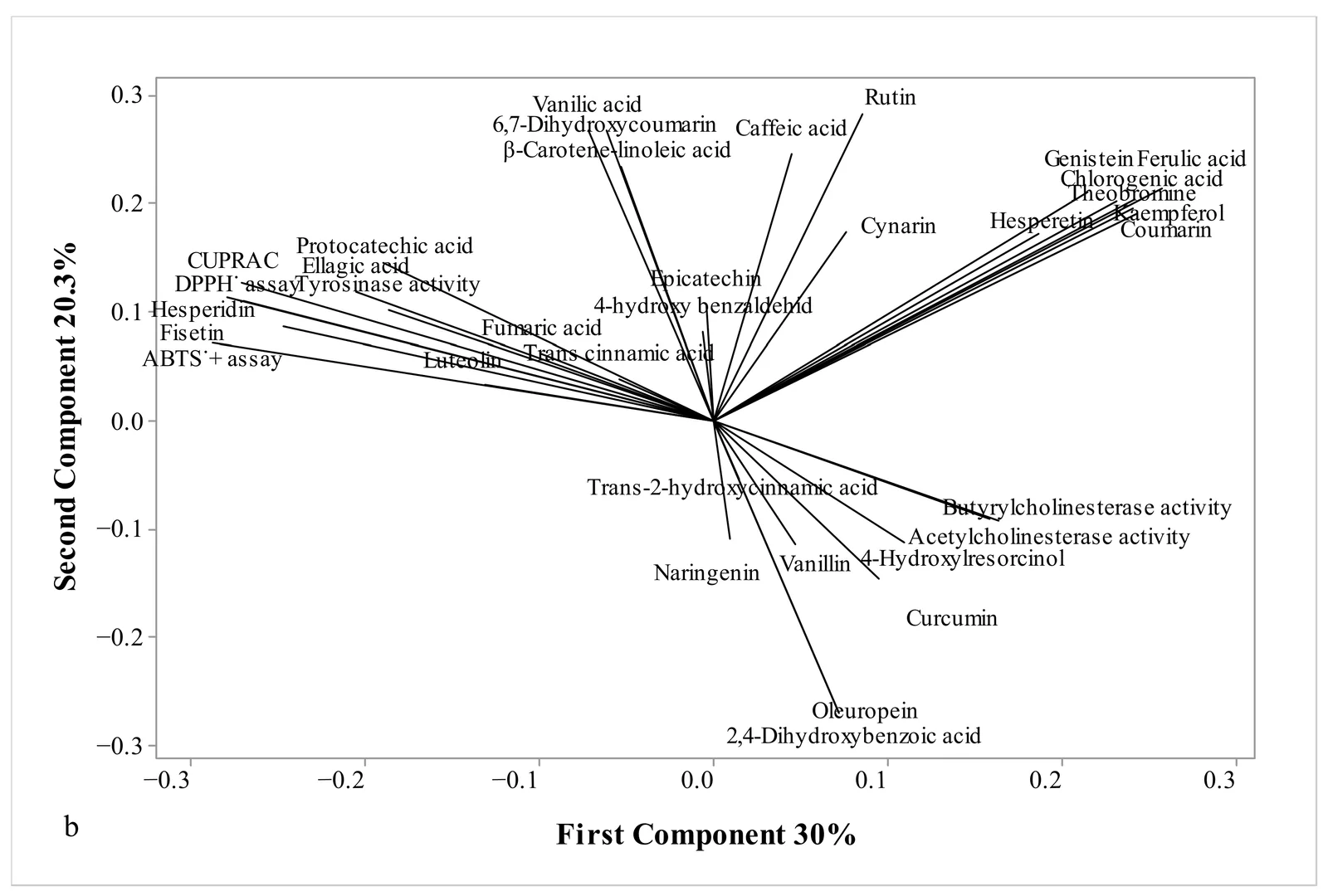
(**a**) Score graph ■ Samples collected in April ● Samples collected in November. (**b**) Loading graph of different solvent extracts of *F. carica* leaves harvested in April and November.

In Figure 2a, the score plot of the principal component analysis (PCA) is presented, while Figure 2b shows the loading plot. Examination of the PCA loading plot revealed that the variables (phenolic compounds and biological activity parameters) were clustered into three characteristic groups.

The samples coded FC14 and FC13, located in the upper-left region of the plot, emerged as the most dominant samples in terms of activities such as CUPRAC, DPPH˙, ABTS˙^+^, and tyrosinase inhibition activity. Evaluation of the score and loading plots together indicated that compounds such as protocatechuic acid, ellagic acid, hesperidin, fisetin, luteolin, fumaric acid, and *trans*-cinnamic acid were also dominant variables in these samples. These results demonstrate that fresh shoot leaves collected in April yielded extracts with the highest antioxidant capacity when processed using water and aqueous ethanol extraction systems. The higher position of FC14 along the PC2 axis compared with FC13 indicates that pure water was able to extract these compounds as effectively as ethanol. In contrast, anticholinesterase activity for this group was positioned in the opposite direction and was relatively low. This observation supports the strong negative correlation between BChE activity and phenolic compounds such as chlorogenic acid, ferulic acid, kaempferol, and rutin, as identified in the Pearson correlation analysis. FC23 and FC24, harvested in November, were also found to be effective to a certain extent with respect to the same compounds and activities, although not as prominently as FC13 and FC14.

The second group consisted of methanol extracts (FC12 and FC22), located in the upper-right region of the plot. Genistein, ferulic acid, chlorogenic acid, theobromine, kaempferol, hesperetin, and coumarin were identified as the dominant variables in these samples. This finding suggests that methanol is the most effective solvent for extracting flavonoids and phenolic acids with moderate to high polarity. FC12 occupied a more extreme position along both principal component axes compared with FC22, indicating that the methanol extract obtained from leaves collected in April was relatively richer in these compounds. Finally, the main discriminating variables of the ethyl acetate extracts (FC11 and FC21) were naringenin, vanillin, curcumin, 2,4-dihydroxybenzoic acid, and oleuropein. This finding suggests that a solvent with moderate to low polarity, such as ethyl acetate, enriches less polar phenolic compounds, and that these extracts particularly stand out in terms of acetylcholinesterase and butyrylcholinesterase inhibition potential. The more negative position of FC21 along the PC2 axis relative to FC11 indicates that this activity may be more pronounced in leaves collected during November.

Table S3 summarizes the loading values, eigenvalues, variance (%), and cumulative variance (%) of different solvent extracts of *F. carica* leaves harvested in April and November, whereas Table S4 presents the score values. As highlighted in bold in Table S3, the variables contributing most positively to the first principal component were theobromine, chlorogenic acid, ferulic acid, coumarin, genistein, and kaempferol. These compounds were detected at the highest levels in sample FC12, as confirmed by the score values highlighted in bold in Table S4. In contrast, the variables contributing negatively to the first principal component were hesperidin, fisetin, ellagic acid, DPPH˙, ABTS˙^+^, and CUPRAC; these variables were found at low levels in FC12 but at the highest levels in FC13 and FC14. The dominant variables of the second principal component were vanillic acid, 6,7-dihydroxycoumarin, ferulic acid, caffeic acid, rutin, genistein, and *β*-carotene–linoleic acid.

Overall evaluation of the PCA results indicated that solvent polarity was a more decisive factor than harvest period in determining the chemical composition and associated biological activities of *F. carica* leaf extracts, as extracts prepared using the same solvent (particularly methanol and ethyl acetate extracts) were positioned closely to each other in the PCA plane. Nevertheless, the harvest period also caused noticeable differentiation, particularly in extracts prepared with polar solvents (water and aqueous ethanol). While the April extracts exhibited superior antioxidant activity, the November extracts displayed a comparatively more moderate profile in terms of these properties.

#### 3.3.3. Hierarchical Cluster Analysis (HCA)

**Figure 3:**
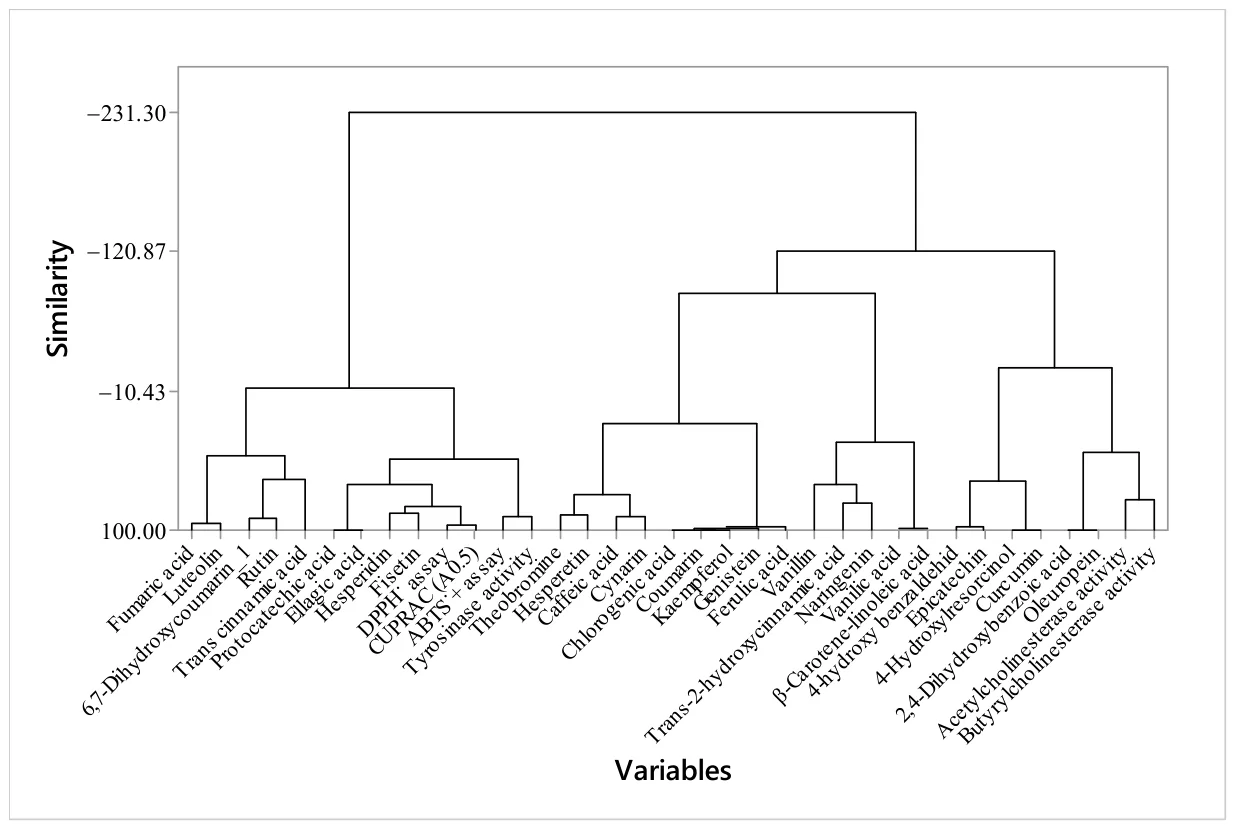
Hierarchical cluster analysis (HCA) dendrogram of *F. carica* leaf extracts obtained using Ward’s method.

Figure 3 presents the dendrogram obtained from hierarchical cluster analysis (HCA) performed using Ward’s method. The dendrogram visually illustrates the similarity relationships among phenolic compounds and biological activity parameters.

At the highest level of the dendrogram (Similarity = −231.30), the variables were divided into two main clusters showing the greatest separation from each other:

Cluster 1 consisted of fumaric acid, luteolin, 6,7-dihydroxycoumarin, rutin, *trans*-cinnamic acid, protocatechuic acid, ellagic acid, hesperidin, fisetin, DPPH˙, CUPRAC, ABTS˙^+^, and tyrosinase activity.

Cluster 2 consisted of theobromine, hesperetin, caffeic acid, cynarin, chlorogenic acid, coumarin, kaempferol, genistein, ferulic acid, vanillic acid, *trans*-2-hydroxycinnamic acid, naringenin, vanillin, *β*-carotene–linoleic acid, 4-hydroxybenzaldehyde, epicatechin, 4-hydroxyresorcinol, curcumin, 2,4-dihydroxybenzoic acid, oleuropein, acetylcholinesterase activity, and butyrylcholinesterase activity.

Within the first cluster, DPPH˙, CUPRAC, ABTS˙^+^, and tyrosinase activity were positioned very close to each other, indicating that these parameters exhibited similar behavior. Compounds clustered near these activities, including fisetin, hesperidin, protocatechuic acid, rutin, *trans*-cinnamic acid, luteolin, fumaric acid, and 6,7-dihydroxycoumarin, showed strong associations with these biological activities. In particular, the grouping of DPPH˙, ABTS˙^+^, and CUPRAC within the same subcluster suggests that these assays similarly reflect antioxidant capacity. Coumarin, kaempferol, genistein, ferulic acid, vanillin, *trans*-2-hydroxycinnamic acid, naringenin, vanillic acid, *β*-carotene–linoleic acid, and 4-hydroxybenzaldehyde were clustered together. This group largely overlaps with the phenolic compounds profile identified as dominant in the methanol extracts (FC12 and FC22). Epicatechin, 4-hydroxyresorcinol, curcumin, 2,4-dihydroxybenzoic acid, oleuropein, acetylcholinesterase activity, and butyrylcholinesterase activity were grouped within the same subcluster. The close clustering of cholinesterase inhibition activities (AChE and BChE), particularly with oleuropein, curcumin, and 2,4-dihydroxybenzoic acid, clarifies the chemical basis of the cholinesterase inhibition potential observed in ethyl acetate extracts (FC11 and FC21) and confirms the PCA results.

Based on the phenolic profiling and statistical analyses, selected compounds were subsequently subjected to molecular docking to provide complementary in silico information on their potential interactions with the investigated enzyme targets.

#### 3.3.4. Molecular Docking Analysis

Molecular docking studies were performed to evaluate the predicted binding affinities of the selected phytochemicals toward the active sites of AChE, BChE, and tyrosinase enzymes, and the corresponding binding energy values are presented in Table 3. Overall, the docking results indicated that several phenolic compounds exhibited favorable binding affinities compared with the reference inhibitors. For AChE, hesperidin showed the strongest predicted interaction, with a binding energy of −9.3 kcal/mol, which was lower than that of the standard inhibitor galantamine (−7.8 kcal/mol). Cynarin (−8.4 kcal/mol), rutin (−8.2 kcal/mol), and ellagic acid (−7.9 kcal/mol) also displayed stronger or comparable binding affinities relative to galantamine, suggesting their potential contribution to AChE inhibitory activity.

In the case of BChE, hesperidin and rutin exhibited the most favorable predicted binding energies, both with values of −10.8 kcal/mol, followed by cynarin (−10.3 kcal/mol), ellagic acid (−9.9 kcal/mol), kaempferol (−9.6 kcal/mol), and epicatechin (−9.1 kcal/mol). These compounds showed more negative binding energies than the reference inhibitor galantamine (−8.9 kcal/mol), indicating comparatively favorable predicted interactions with the BChE active site. Naringenin displayed a binding energy of −8.8 kcal/mol, which was close to that of galantamine, whereas chlorogenic acid showed a moderately favorable binding energy of −8.3 kcal/mol. In contrast, ferulic acid (−6.8 kcal/mol), coumarin (−6.5 kcal/mol), 4-hydroxyresorcinol (−5.9 kcal/mol), and fumaric acid (−5.0 kcal/mol) exhibited weaker predicted interactions. Overall, the docking results suggest that flavonoid glycosides and related polyphenolic compounds, particularly hesperidin, rutin, cynarin, ellagic acid, and kaempferol, may contribute to the predicted BChE-binding potential of the selected *F. carica* constituents.

A similar pattern was observed for tyrosinase, where most of the tested compounds displayed stronger predicted binding affinities than kojic acid, the standard tyrosinase inhibitor. Rutin showed the strongest binding energy against tyrosinase (−10.3 kcal/mol), followed by hesperidin (−10.1 kcal/mol), cynarin (−9.4 kcal/mol), kaempferol (−9.1 kcal/mol), naringenin and chlorogenic acid (−8.9 kcal/mol), epicatechin (−8.3 kcal/mol), and ellagic acid (−8.2 kcal/mol). In contrast, fumaric acid (−4.4 kcal/mol) and 4-hydroxyresorcinol (−5.2 kcal/mol) exhibited weaker interactions relative to kojic acid (−5.5 kcal/mol). Overall, the docking results support the possible involvement of hesperidin, rutin, cynarin, ellagic acid, kaempferol, naringenin, and chlorogenic acid in the enzyme inhibitory effects of *F. carica* leaf extracts. However, these findings should be interpreted as preliminary in silico evidence and require further experimental validation.

In silico analyses demonstrate that hesperidin displays favorable binding affinity values toward AChE, BChE, and tyrosinase. Similarly, the rutin compound also exhibits high binding energy values against the AChE, BChE, and tyrosinase enzymes. Such binding energy magnitudes are noteworthy for a naturally occurring compound and imply significant potential for biological activity. Protein–ligand molecular docking analyses represent a key computational approach in modern drug discovery.

**Figure 4:**
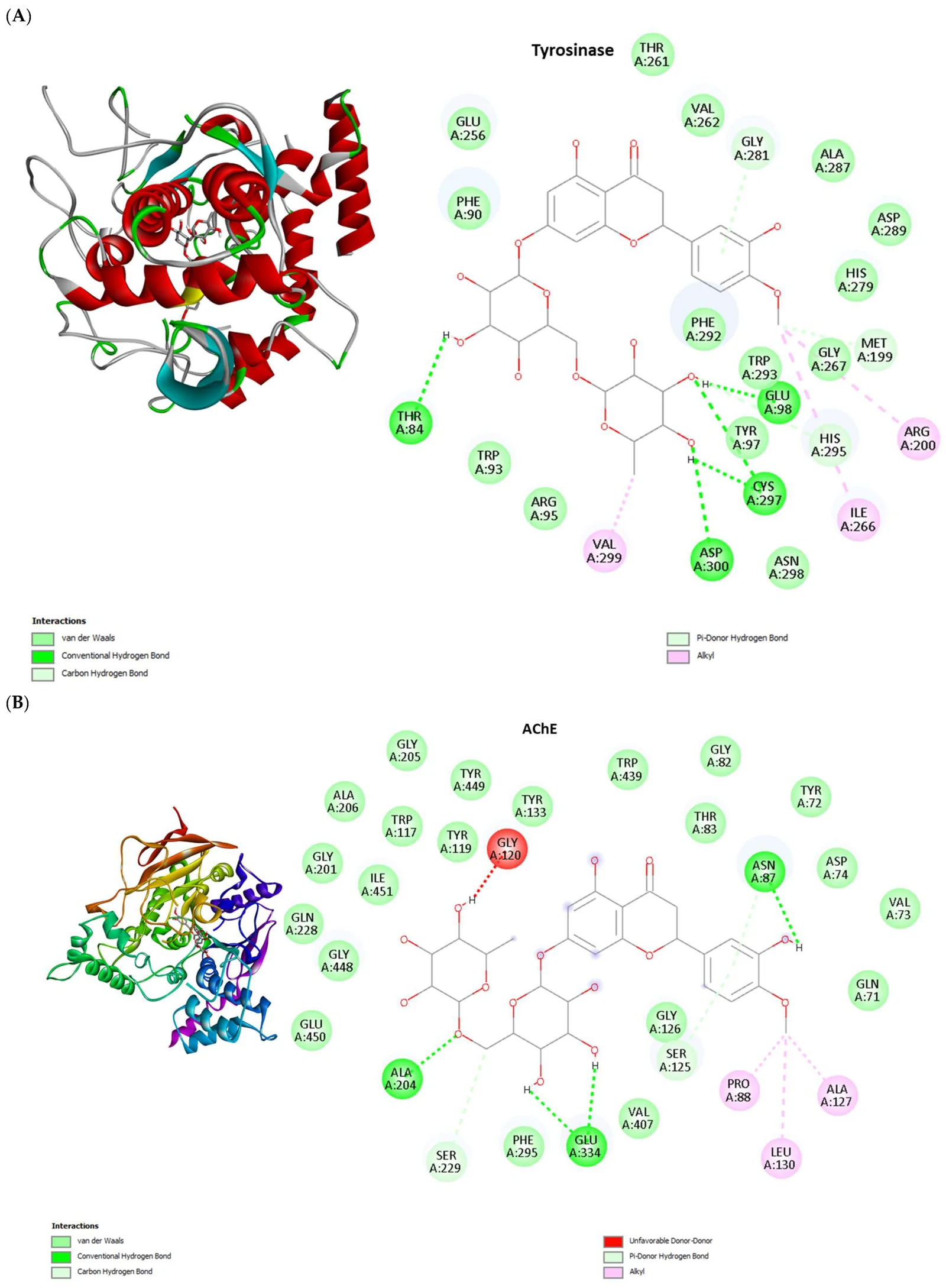

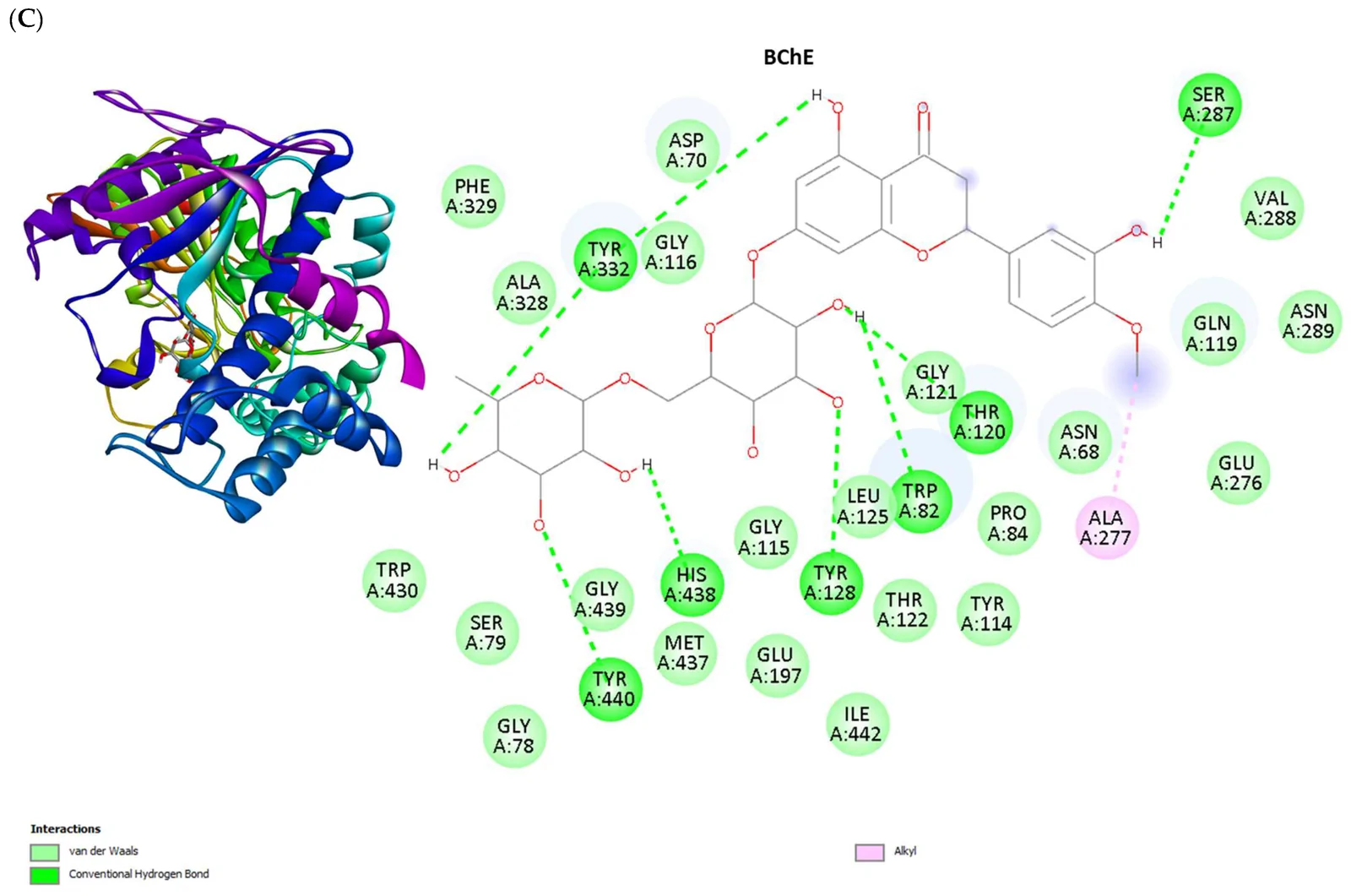
Molecular docking interactions of hesperidin within the catalytic pockets of tyrosinase (**A**), AChE (**B**) and BChE (**C**). AChE, acetylcholinesterase; BChE, butyrylcholinesterase.

**Figure 5:**
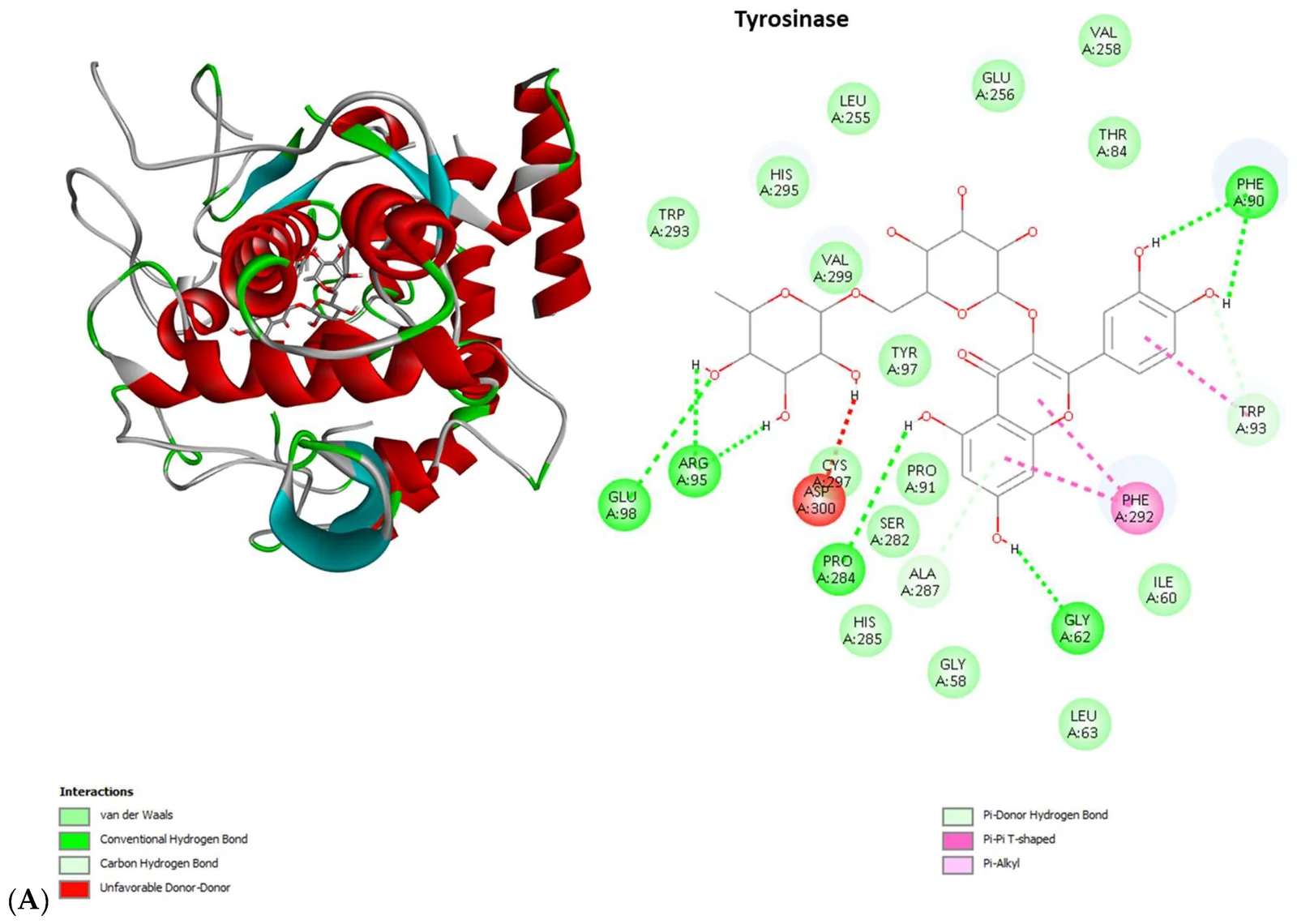

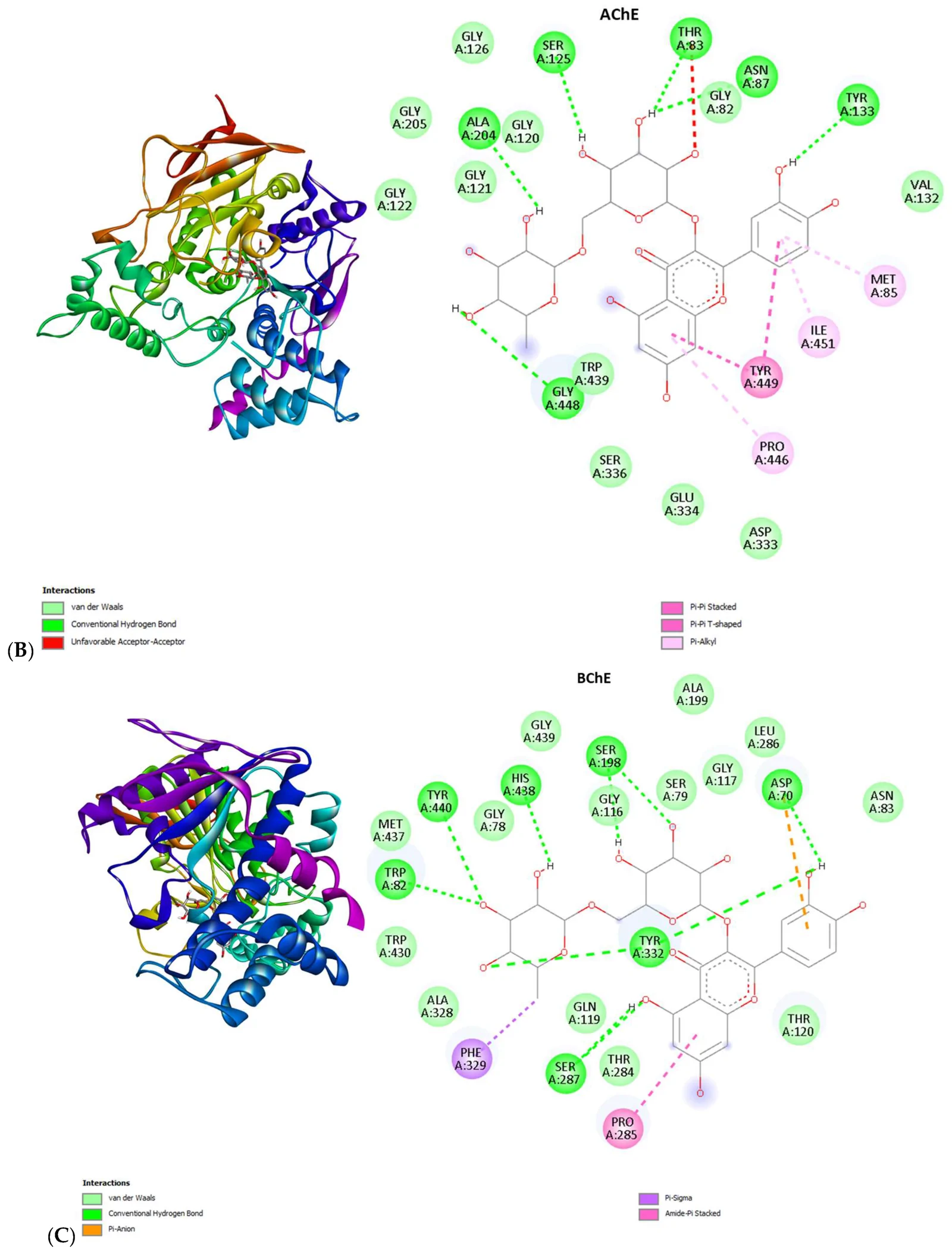
Molecular docking interactions of rutin within the catalytic pockets of tyrosinase (**A**), AChE (**B**) and BChE (**C**). AChE, acetylcholinesterase; BChE, butyrylcholinesterase.

Docking methodologies are widely applied in pharmaceutical research, particularly for virtually screening extensive chemical libraries to identify potential therapeutic candidates. Complementing experimental efforts aimed at elucidating ligand–binding mechanisms, a docking investigation was performed for selected phytochemical constituents identified in *F. carica* leaf extracts. Representative binding poses and two-dimensional interaction maps of hesperidin and rutin within the active sites of AChE, BChE, and tyrosinase are presented in Figure 4 and Figure 5, respectively.

The corresponding docking interaction diagrams for the remaining compounds are provided in Figure S9 in the Supplementary Information. Hesperidin is a natural compound with a flavanone glycoside structure, and due to its aromatic rings and numerous hydroxyl groups, it can interact with biological targets with high specificity.

In this analysis, the molecular binding behavior of the hesperidin molecule with the AChE (acetylcholinesterase) enzyme was evaluated, and the interactions were classified based on conventional hydrogen bonds, hydrophobic contacts, and van der Waals interactions. Hesperidin establishes binding stability in the active site of the AChE enzyme by forming hydrogen bonds through its glycosyl and flavonoid hydroxyl groups with the Ala204, Glu334, and Asn87 residues. Alkyl interactions were identified between the aromatic core of hesperidin and the aliphatic and aromatic side chains of the enzyme, specifically involving the Pro88, Leu130, and Ala127 residues. Due to its large molecular volume, hesperidin forms extensive van der Waals interactions with numerous amino acid residues within the binding pocket, including Ala206, Gly126, Gly201, Gly205, Gly448, Gln228, Glu450, Tyr449, Tyr133, Tyr119, Thr83, Val407, Trp117, Ile451, Gly82, Tyr72, Asp74, Val73, Gln71, Trp439, and Phe295.

This large number of close contacts demonstrates additional hydrophobic and steric complementarity, which contributes to enhancing the overall binding strength and stability. The binding of the ligand to this enzyme occurs through a mixed mechanism involving both polar hydrogen bonds and hydrophobic alkyl interactions. Phe295 is one of the residues implicated in the activity of AChE, which participates in the formation of the acyl pocket subsite [59].

Molecular docking analyses within the BChE active site demonstrate the occurrence of characteristic polar interactions and hydrogen bonding. Owing to variations in the amino acid composition of the BChE binding pocket, the interaction pattern between inhibitor molecules and BChE differs from that observed in their interactions with AChE [60]. Hesperidin formed hydrogen bonds with several amino acid residues within the BChE binding site, including Ser287, Thr120, Tyr332, Trp82, Tyr128, His438, and Tyr440. These hydrogen bonds are widely distributed across both the aromatic core and the glycosyl chain of the molecule. The phenyl ring of the ligand establishes a specific alkyl interaction with the Ala277 residue. The ligand extends over a wide area within the binding pocket, forming van der Waals contacts with numerous amino acid residues, including Ala328, Phe329, Asp70, Gly116, Asn289, Gly115, Gly78, Gln119, Glu276, Asn68, Gly121, Val288, Met437, Ile442, Glu197, Tyr114, Thr122, Trp430, Pro84, Leu125, and Ser79. The ligand is anchored within the BChE active site through multiple strong hydrogen bonds, while simultaneously maintaining hydrophobic interactions via alkyl contacts.

The hesperidin molecule forms hydrogen bonds through its hydroxyl groups with Asn57 and Met61 residues within the active site of the tyrosinase enzyme. Additionally, a hydroxyl group on the ligand’s phenyl ring forms a hydrogen bond with the Leu66 and Arg70 residues. The aromatic rings of hesperidin establish alkyl system interactions with the aromatic residues of tyrosinase, including Trp238, Pro222, Val234, and Leu73. These interactions indicate that the aromatic portions of the ligand are well accommodated within the hydrophobic and steric pockets of the tyrosinase active site. Hesperidin engages in Van der Waals interactions with multiple residues within the tyrosinase active site, including Phe228, Tyr72, Trp68, Gly46, Arg206, Arg209, Gly216, Val207, Ala232, His230, Met215, Asp235, and Ala59. When analyzing hesperidin binding to the three enzymes, it is evident that the glycosyl moiety primarily engages in hydrogen bonding, while the flavonoid core contributes through hydrophobic interactions as the main binding strategy. However, the quantity and nature of these interactions vary depending on the specific enzyme. BChE stands out as the enzyme with the highest polar affinity, forming the greatest number and most widely distributed hydrogen bonds with hesperidin. This dense hydrogen bonding network indicates that the polar regions of the ligand fit tightly and specifically into the active site of BChE. This suggests that BChE exhibits the highest potential affinity for hesperidin, as evidenced by the extensive and well-distributed hydrogen bond interactions (Table 3).

The interaction with AChE exhibits a balance between polar and hydrophobic interactions; however, it is not as extensive as that observed with BChE. Although it forms fewer hydrogen bonds compared to BChE, the extensive alkyl interactions established by the flavonoid core make a significant contribution to hydrophobic binding. The interaction with tyrosinase is characterized by strong hydrophobic accommodation driven by aromatic π-system stacking. This indicates that the aromatic rings of the flavonoid are strongly anchored within the hydrophobic pockets of the tyrosinase active site.

Rutin binds to all three enzymes through extensive polar, aromatic, and hydrophobic interactions, facilitated by its rutinoside moiety and quercetin core. The rutin molecule exhibits high binding stability by forming multiple hydrogen bonds with amino acid residues located in the active site of AChE, including Ser125, Thr83, Asn87, Ala204, Tyr133, and Gly448.

Most of these hydrogen bonds are established between the –OH groups located on the glycosyl moiety of the molecule and the side chains of the amino acid residues. Rutin binds to the enzyme surface with a remarkably large contact area, establishing van der Waals (vdW) interactions with the amino acid residues Gly120, Gly122, Gly205, Gly126, Gly82, Val132, Asp333, Glu334, Trp439, and Ser336. The flavonoid core of the ligand establishes a strong and stabilizing π–π stacking interaction with the aromatic ring of the Tyr449 residue located in the enzyme’s active site. This interaction provides the most significant contribution to the binding affinity. The aromatic rings of rutin engage in alkyl interactions with the enzyme’s aromatic and hydrophobic regions, particularly with residues Pro446, Ile451, and Met85. These bonds are primarily formed around the flavonoid core of the molecule and play a pivotal role in significantly enhancing its binding affinity to the enzyme. Rutin exhibits a binding affinity to the active site of the AChE enzyme through a combination of hydrogen bonds, as well as aromatic and hydrophobic interactions. The presence of numerous hydrogen bonds, along with π–π stacking and π–alkyl interactions, clearly indicates that rutin exhibits a high affinity toward the enzyme.

Rutin is a natural flavonoid glycoside that, due to its multiple hydroxyl groups and aromatic rings, can bind to biological targets with high affinity. Molecular docking analysis was conducted to evaluate the interaction profile of the rutin molecule within the active site of the BChE (butyrylcholinesterase) enzyme. The abundance of hydroxyl groups in the rutin molecule facilitates the formation of hydrogen bonds with key residues in the enzyme’s active site, such as Ser198, Ser287, His438, Tyr440, Trp82, Tyr332, and Asp70. The aromatic rings of rutin establish π-based interactions with the enzyme’s aromatic residues, further enhancing its binding energy. In this context, a π–sigma interaction was formed with the Phe329 residue, while an amide–π stacked interaction occurred with Pro285. Additionally, rutin established a π–anion interaction with the Asp70 residue. The delocalized π-electron cloud of the flavonoid ring contributes to binding stability by generating an electrostatic attraction with the negatively charged carboxylate group.

The broad molecular surface of rutin facilitates strong van der Waals interactions with numerous amino acid residues within the active site of the enzyme, including Trp430, Met437, Gly78, Gly439, Gly116, Ser79, Gly117, Gln119, Ala328, Thr120, Thr284, Leu286, Asn83, and Ala199. Rutin exhibits a stable and versatile binding behavior toward the active site of butyrylcholinesterase (BChE), engaging through multiple interaction types. In addition to hydrogen bonding, the presence of aromatic system-specific interactions such as π–anion, π–sigma, and amide–π stacked indicates that the binding is not limited to hydrophilic contacts alone.

As a natural flavonoid glycoside, rutin binds to the binding site of the tyrosinase enzyme through its structure rich in hydroxyl groups and aromatic rings. Owing to its numerous free –OH groups, rutin forms conventional hydrogen bonds with several residues, particularly Glu98, Arg95, Pro284, Gly62, and Phe90. These interactions indicate that the hydroxyl-rich glycosyl and flavonoid portions of rutin make an important contribution to the polar stabilization of the ligand within the binding pocket. The aromatic rings of rutin also participate in π-based interactions within the tyrosinase binding site. In particular, π–π T-shaped interactions are observed with Phe292 and Trp93, indicating additional stabilization of the aromatic scaffold within the binding pocket. The ligand is further surrounded by several residues contributing mainly through van der Waals interactions, including His295, Trp293, Leu255, Glu256, Val258, Thr84, Val299, Tyr97, Pro91, Ser282, Ala287, His285, Gly58, Leu63, and Ile60. Ala328 has been reported as a catalytically important residue for BChE based on mutagenesis studies [60]. In the present docking analysis, hesperidin and rutin formed multiple hydrogen-bonding and other noncovalent interactions with residues located within the respective enzyme-binding pockets. These active site constituent residues are very close to the catalytically essential residues known from the literature [61].

### 3.4. İn Silico Physicochemical, Pharmacokinetic, and Toxicity Prediction

The physicochemical, pharmacokinetic, and toxicity profiles of the selected bioactive phenolic compounds were evaluated to support the molecular docking findings and to obtain preliminary information on their drug-likeness and safety-related properties. The detailed prediction data are presented in Tables S5–S7.

As shown in Table S5, the selected compounds exhibited different physicochemical and drug-likeness profiles. Hesperidin and rutin had relatively high molecular weights, 610.56 and 610.52 g/mol, respectively, along with high TPSA values of 234.29 and 269.43 Å^2^. Both compounds showed three Lipinski rule-of-five violations and low predicted gastrointestinal absorption, indicating limited oral drug-likeness. Similarly, cynarin showed a high molecular weight of 516.45 g/mol, a TPSA value of 211.28 Å^2^, and three Lipinski violations, suggesting reduced oral absorption potential. Chlorogenic acid displayed one Lipinski violation and low predicted gastrointestinal absorption. In contrast, ellagic acid, kaempferol, naringenin, and ferulic acid complied with Lipinski’s rule of five without any violations and showed high predicted gastrointestinal absorption. Among these compounds, ferulic acid exhibited the most favorable bioavailability score of 0.85, whereas ellagic acid, kaempferol, and naringenin showed bioavailability scores of 0.55.

The pharmacokinetic prediction results presented in Table S6 further support the physicochemical findings. Ferulic acid, naringenin, ellagic acid, and kaempferol showed the highest predicted intestinal absorption values, with 93.685%, 91.310%, 86.684%, and 74.290%, respectively. These results may be associated with their lower molecular weights, lower TPSA values, and absence of Lipinski violations. By contrast, hesperidin, rutin, chlorogenic acid, and cynarin showed relatively lower intestinal absorption values ranging from 23.446% to 36.377%, probably due to their higher polarity, greater hydrogen-bonding capacity, and larger molecular size. This interpretation is consistent with human pharmacokinetic studies showing that hesperidin and rutin are not predominantly detected in circulation as unchanged glycosides but are converted to hesperetin- and quercetin-derived conjugates, respectively. The absorption of these compounds is also influenced by intestinal hydrolysis and colonic microbial metabolism.

Chlorogenic acid showed low GI absorption in SwissADME and a predicted intestinal absorption of 36.377% in pkCSM. This value agrees closely with the approximately 33% absorption reported in human volunteers. Nevertheless, chlorogenic acid undergoes substantial hydrolysis and microbial metabolism, and its biological effects may therefore involve caffeic acid, ferulic acid, and other downstream metabolites rather than exclusively the unchanged parent compound [62,63].

Regarding permeability, naringenin showed the highest predicted Caco-2 permeability value, followed by hesperidin, ellagic acid, ferulic acid, and kaempferol, whereas rutin, chlorogenic acid, and cynarin displayed lower predicted permeability. P-glycoprotein substrate behavior was predicted for hesperidin, cynarin, and naringenin, suggesting that active efflux mechanisms may influence their absorption and tissue distribution [64].

Ferulic acid and naringenin exhibited the most favorable predicted absorption profiles, with intestinal absorption values of 93.685% and 91.310%, respectively. Naringenin was also the only compound with a pkCSM Caco-2 permeability value above the high-permeability threshold of 0.90. However, high intestinal absorption should not be equated with high systemic bioavailability of the unchanged compound. Human pharmacokinetic studies have demonstrated extensive first-pass conjugation of naringenin, while ferulic acid is rapidly converted to glucuronide and sulfate metabolites after absorption. Indeed, ferulic acid is rapidly absorbed from the gastrointestinal tract; however, it undergoes extensive presystemic metabolism, particularly conjugation in the liver It has beenreported that free ferulic acid represented approximately 49% of total ferulic acid in portal venous plasma, whereas this proportion decreased to only 5–8% in systemic arterial plasma, indicating extensive hepatic conjugation before reaching the systemic circulation. Therefore, although the present pkCSM analysis predicted a high intestinal absorption of ferulic acid (93.685%), this value represents predicted intestinal absorption and should not be interpreted as systemic bioavailability of the unchanged compound. The extensive first-pass metabolism of ferulic acid may substantially limit systemic exposure to its unmetabolized form [65].

Kaempferol was predicted to have high GI absorption and moderate-to-high intestinal absorption. Human studies support its gastrointestinal uptake; however, kaempferol circulates predominantly as glucuronide and sulfate conjugates. Similarly, although ellagic acid fulfilled Lipinski’s criteria and was classified as having high GI absorption, its low predicted aqueous solubility and experimentally reported low plasma exposure indicate that dissolution, presystemic metabolism, and conversion to urolithins may substantially restrict its actual oral bioavailability.

The BBB predictions should also be interpreted cautiously. Hesperidin, rutin, chlorogenic acid, cynarin, and ellagic acid showed pkCSM log BB values below −1, indicating poor predicted brain distribution [66]. In contrast, naringenin, kaempferol, and ferulic acid showed intermediate values, although only ferulic acid was classified as BBB-permeant by SwissADME. Thus, the strong docking affinities of highly polar glycosides toward AChE and BChE should not be considered direct evidence of central nervous system activity, as their access to the brain may be limited. The pkCSM analysis predicted cynarin as a CYP3A4 substrate rather than a CYP3A4 inhibitor. Ellagic acid, kaempferol, and naringenin were predicted to inhibit CYP1A2, suggesting that these compounds may warrant further experimental investigation for potential metabolic interactions. However, in silico CYP predictions alone are insufficient to establish clinically relevant herb–drug interactions and should be confirmed using enzyme inhibition assays.

Overall, ferulic acid, naringenin, and kaempferol demonstrated comparatively favorable predicted physicochemical and intestinal absorption properties. Nevertheless, the systemic exposure of the unchanged parent compounds may remain limited because of poor aqueous solubility, intestinal transformation, active efflux, and extensive phase II metabolism. Accordingly, the present ADME results should be regarded as preliminary structure-based predictions and should be interpreted together with experimental pharmacokinetic data.

Toxicity prediction is an important step in the early stage evaluation of bioactive compounds, as promising drug candidates should exhibit not only favorable biological activity but also an acceptable safety profile. In this study, the toxicity profiles of eight selected bioactive phenolic compounds identified in *F. carica* leaf extracts were predicted using the ProTox-II online server. The evaluated parameters included predicted LD_50_ values, toxicity class, hepatotoxicity, carcinogenicity, immunotoxicity, mutagenicity, and cytotoxicity, as presented in Table S7. In the pkCSM model, all selected compounds were predicted to be negative for AMES toxicity, hepatotoxicity, and skin sensitization.

The predicted acute oral LD_50_ values ranged from 1772 to 12,000 mg/kg. Hesperidin was assigned to toxicity class VI, whereas rutin, chlorogenic acid, cynarin, kaempferol, and ellagic acid were classified as toxicity class V. Ellagic acid should be classified as class V rather than class IV because its predicted LD_50_ value of 2991 mg/kg lies within the 2000–5000 mg/kg interval. Naringenin and ferulic acid were classified as class IV. These classifications suggest relatively low predicted acute oral toxicity compared with highly toxic chemicals; however, they should not be interpreted as evidence of overall safety.

Moreover, the hepatotoxicity, carcinogenicity, immunotoxicity, mutagenicity, and cytotoxicity outputs of ProTox-II should be reported as predicted active or inactive classifications together with their confidence probabilities. Probability values alone cannot establish whether an endpoint was predicted to be toxic or non-toxic. Consequently, the toxicity results should be considered preliminary computational alerts that require confirmation by appropriate in vitro and in vivo studies.

## 4. Conclusions

In this study, *Ficus carica* leaves collected from Muğla were evaluated in this context for the first time. The in vitro findings were further corroborated by in silico analyses, which indicated that these compounds possess strong enzyme inhibition potential. The biological effects observed in *F. carica* appear to be largely attributable to its major constituents, particularly hesperidin and rutin. Molecular docking analyses identified the binding interactions and affinities of the compounds with AChE, BChE, and tyrosinase. Interestingly, all tested molecules showed effective interactions with these enzymes. İn silico findings further demonstrated that hesperidin and rutin bind to the active sites of the target enzymes with high specificity and have the potential to exert inhibitory effects through various interactions with key amino acid residues. Pearson correlation analysis indicated that several phenolic compounds, including chlorogenic acid, ferulic acid, kaempferol, and rutin, exhibited strong negative correlations with BChE activity, suggesting their potential contribution to enzyme inhibition. In contrast, compounds such as hesperidin, fisetin, and ellagic acid showed positive correlations with tyrosinase inhibition, indicating their possible role in modulating enzyme activity through different mechanisms. The HCA results support the PCA findings, indicating that the biological activities of *F. carica* leaf extracts are closely associated with specific groups of phenolic compounds. The dendrogram revealed a clear distinction between antioxidant activity and phenolic acids, as well as between anticholinesterase activity and less polar phenolic compounds. Overall, these findings demonstrate that the biological activity of the extracts is closely related to their phenolic profile, and specific compounds may act either as enhancers or modulators of enzyme inhibition. This highlights the importance of phenolic diversity in determining the functional and therapeutic potential of plant-derived extracts. Owing to their diverse chemical profiles and notable bioactivities, *F. carica* species represent a promising source of natural bioactive agents with potential applications in the food, cosmetic, and pharmaceutical sectors. Although most research on *F. carica* remains limited to in vitro and pre-clinical studies, additional clinical and human trials are required before it can be fully integrated into clinical practice or personalized nutrition strategies. However, despite their strong docking performance, hesperidin and rutin showed limited predicted oral drug-likeness, indicating that further pharmacokinetic validation or formulation-based approaches may be required to support their potential bioactive applications. However, it should be acknowledged as a limitation that the plant material was gathered from a single individual tree, which may constrain the broader generalization of these observed seasonal differences to the entire *F. carica* population.

Overall, the present results demonstrate that harvesting period and extraction solvent significantly influenced the phenolic composition, antioxidant capacity, and enzyme inhibitory activity of *F. carica* leaf extracts. Among the investigated samples, water and aqueous ethanol extracts showed pronounced antioxidant activity, whereas the November water extract exhibited the strongest AChE and tyrosinase inhibitory activities. Hesperidin, rutin, chlorogenic acid, cynarin, naringenin, and other phenolic constituents showed associations with the observed bioactivities based on chemometric analyses. Molecular docking provided complementary information on the possible interactions of selected compounds with AChE, BChE, and tyrosinase, while ADMET analyses provided preliminary predictions of their pharmacokinetic and safety-related properties. However, these in silico findings require experimental validation, and the biological activities observed for the extracts should not be attributed to individual compounds without further targeted studies.

## Figures and Tables

**Table 1:** HPLC-DAD profiles of the methanol, aqueous ethanol, and water extracts of *F. carica* leaves harvested in April and November (mg/g).

Compounds	FC11	FC12	FC13	FC14	FC21	FC22	FC23	FC24
Fumaric acid	−	−	1.74 ± 0.01 ^A^	−	−	0.80 ± 0.05 ^B^	−	−
Protocatechuic acid	0.03 ± 0.0.01 ^D^	0.08 ± 0.005 ^B^	0.05 ± 0.005 ^C,D^	0.43 ± 0.01 ^A^	*tr*	0.07 ± 0.001 ^B,C^	0.09 ± 0.001 ^B^	0.04 ± 0.001 ^D^
Theobromine	−	*tr*	−	−	−	*tr*	−	−
4-hydroxybenzoic acid	−	−	−	0.01 ± 0.001 ^A^	−	−	−	−
6,7-dihydroxycoumarin	−	*tr*	*tr*	*tr*	−	*tr*	−	*tr*
4-hydroxybenzaldehyde	*tr*	0.01 ± 0.001 ^B^	*tr*	*tr*	−	0.18 ± 0.01 ^A^	−	−
Vanillic acid	*tr*	*tr*	*tr*	*tr*	−	*tr*	*tr*	*tr*
Caffeic acid	−	*tr*	−	*tr*	−	*tr*	*tr*	−
Epicatechin	−	−	−	0.56 ± 0.01 ^B^	−	1.72 ± 0.05 ^A^	−	−
Vanillin	*tr*	−	−	−	−	−	−	−
Chlorogenic acid	0.31 ± 0.01 ^E^	8.99 ± 0.07 ^A^	0.18 ± 0.01 ^G^	0.50 ± 0.01 ^D^	0.28 ± 0.01 ^E,F^	0.69 ± 0.01 ^C^	0.91 ± 0.01 ^B^	0.22 ± 0.01 ^F,G^
2,4-dihydroxy benzoic acid	−	−	−	−	*tr*	−	−	−
Ferulic acid	*tr*	1.90 ± 0.03 ^A^	*tr*	−	−	0.49 ± 0.01 ^B^	−	−
Cynarin	5.89 ± 0.1 ^D^	5.89 ± 0.08 ^D^	−	5.95 ± 0.1 ^C^	−	6.23 ± 0.2 ^B^	6.33 ± 0.11 ^A^	−
Coumarin	−	0.07 ± 0.01 ^A^	−	−	−	−	−	−
Hesperidin	−	*tr*	39.52 ± 0.03 ^C^	80.06 ± 0.22 ^A^	−	*tr*	−	42.17 ± 0.32 ^B^
Rutin	*tr*	2.91 ± 0.1 ^A^	2.15 ± 0.05 ^C^	1.23 ± 0.01 ^D^	*tr*	2.45 ± 0.1 ^B^	0.22 ± 0.05 ^F^	0.69 ± 0.01 ^E^
*Trans*-2-hydroxycinnamic acid	*tr*	−	−	*tr*	−	*tr*	*tr*	−
Ellagic acid	−	−	−	3.72 ± 0.1 ^A^	−	−	−	−
Fisetin	−	−	0.51 ± 0.01 ^A^	0.28 ± 0.01 ^B^	−	−	−	0.15 ± 0.001 ^C^
Oleuropein	−	−	−	−	0.59 ± 0.01 ^A^	−	−	−
*Trans* cinnamic acid	−	*tr*	*tr*	*tr*	*tr*	−	−	*tr*
Naringenin	2.44 ± 0.21 ^B^	−	−	0.34 ± 0.01 ^D^	0.97 ± 0.01 ^C^	−	4.46 ± 0.03 ^A^	−
Hesperetin	−	*tr*	−	−	−	*tr*	*tr*	−
Genistein	*tr*	*tr*	−	−	−	−	−	−
Luteolin	−	−	0.89 ± 0.08 ^A^	−	−	−	−	−
Kaempferol	−	1.53 ± 0.1 ^A^	−	−	*tr*	−	−	−
4-hydroxylresorcinol	*tr*	1.47 ± 0.1 ^D^	*tr*	*tr*	18.94 ± 0.6 ^B^	25.17 ± 0.41 ^A^	5.71 ± 0.1 ^C^	0.36 ± 0.01 ^E^
Curcumin	−	*tr*	*tr*	*tr*	2.38 ± 0.1 ^B^	2.59 ± 0.22 ^A^	0.96 ± 0.01 ^C^	*tr*

Values are expressed as mean ± SD of three independent extraction replicates (*n* = 3) Different superscript letters in the same row indicate statistically significant differences among the extracts according to one-way ANOVA followed by Tukey’s post-hoc test (*p* < 0.05). ‘*tr*’ indicates trace amounts (below quantification limit but above detection limit), and ‘−’ indicates not detected.

**Table 2:** IC_50_ values (200 μL) of antioxidant and enzyme inhibitory activities of *F. carica* leaf extracts.

	Antioxidant Activity				Enzyme Inhibitory Activity		
Codes	DPPH˙	ABTS˙^+^	*β*-Carotene–Linoleic Acid	CUPRAC (A_0.5_)	Acetylcholinesterase Activity	Butyrylcholinesterase Activity	Tyrosinase Activity
FC11	>200	>200	37.77 ± 2.11 ^F^	>200	153.76 ± 1.15 ^A^	>200	*NA*
FC12	>200	184.66 ± 0.17 ^A^	81.41 ± 0.95 ^B^	>200	>200	181.02 ± 0.34 ^A^	*NA*
FC13	57.54 ± 0.02 ^B^	63.50 ± 0.66 ^D^	65.23 ± 0.76 ^D^	148.08 ± 1.11 ^B^	>200	>200	195.77 ± 2.09 ^A^
FC14	54.46 ± 0.94 ^C^	10.75 ± 0.17 ^G^	125.81 ± 0.23 ^A^	95.38 ± 1.69 ^C^	*NA*	*NA*	>200
FC21	>200	131.18 ± 0.15 ^B^	*NA*	>200	>200	>200	*NA*
FC22	>200	96.75 ± 0.03 ^C^	75.53 ± 0.99 ^C^	>200	>200	*NA*	38.17 ± 0.97 ^C^
FC23	114.98 ± 0.07 ^A^	59.88 ± 1.48 ^F^	73.53 ± 0.55 ^C^	165.05 ± 1.42 ^A^	*NA*	*NA*	88.57 ± 1.46 ^B^
FC24	>200	61.35 ± 0.27 ^E^	48.39 ± 1.54 ^E^	>200	135.52 ± 0.77 ^B^	*NA*	34.46 ± 1.05 ^C^
BHA *	5.09 ± 0.22	3.46 ± 0.34	22.12 ± 0.79	6.14 ± 0.13	*NT*	*NT*	*NT*
Galantamine *	*NT*	*NT*	*NT*	*NT*	9.02 ± 0.81	3.62 ± 0.34	*NT*
Kojic acid *	*NT*	*NT*	*NT*	*NT*	*NT*	*NT*	24.99 ± 0.13

BHA: Butyl Hydroxy Anisole. * Standard compounds. *NA*: not active, *NT*: not tested. Values are expressed as mean ± SD of three independent extraction replicates (*n* = 3). Different superscript letters in the same row indicate statistically significant differences among the extracts according to one-way ANOVA followed by Tukey’s post-hoc test (*p* < 0.05).

**Table 3:** Molecular Docking Analysis of Compounds with the AChE, BChE and Tyrosinase Enzymes for Binding Energy (kcal/mol).

Compounds	AChE	BChE	Tyrosinase
4-hydroxyresorcinol	−5.10	−5.9	−5.40
Chlorogenic Acid	−7.4	−8.3	−8.6
Coumarin	−6.0	−6.5	−6.6
Cynarin	−8.4	−10.3	−8.8
Ellagic Acid	−7.9	−9.9	−8.3
Epicatechin	−7.2	−9.1	−8.2
Ferulic Acid	−5.9	−6.8	−6.5
Fumaric Acid	−4.6	−5.0	−4.8
Hesperidin	−9.3	−10.8	−10.2
Kaempferol	−7.7	−9.6	−8.6
Naringenin	−7.7	−8.8	−8.7
Rutin	−8.2	−10.8	−10.4
Galantamine	−7.8	−8.9	-
Kojic acid	-	-	−5.4

## Data Availability

The data presented in this study are available on request from the corresponding author.

## Supplementary Materials

The following supporting information can be downloaded at: https://www.mdpi.com/article/10.3390/life16091518/s1, Figure S1: HPLC-DAD chromatogram of the FC11 extract; Figure S2: HPLC-DAD chromatogram of the FC21 extract; Figure S3: HPLC-DAD chromatogram of the FC12 extract; Figure S4: HPLC-DAD chromatogram of the FC22 extract; Figure S5: HPLC-DAD chromatogram of the FC13 extract; Figure S6: HPLC-DAD chromatogram of the FC23 extract; Figure S7: HPLC-DAD chromatogram of the FC14 extract; Figure S8: HPLC-DAD chromatogram of the FC24 extract; Figure S9: Docking results of ligands in catalytic pocket of enzymes for compounds. AChE, acetylcholinesterase; BChE, butyrylcholinesterase. Table S1: Gradient elution program used for HPLC analysis; Table S2: Retention time, calibration curves, regression coefficient (R2), linearity ranges, LODs, and recoveries of phenolic standards at 254 nm; Table S3: Loading, Eigenvalue, Variance (%), and Cumulative Variance (%) Values of Different Solvent Extracts of *F. carica* Leaves Harvested in April and November; Table S4: The Score Values of Different Solvent Extracts of *F. carica* Leaves Harvested in April and November; Table S5: Predicted Physicochemical and Drug-Likeness Properties of the Selected Bioactive Phenolic Compounds from *F. carica* Leaf Extracts; Table S6: Predicted pharmacokinetic and toxicity-related properties of the selected bioactive phenolic compounds identified in *F. carica* leaf extracts using pkCSM; Table S7: Predicted Toxicity Profiles of Selected Bioactive Phenolic Compounds from *F. carica* Leaf Extracts Using ProTox-II.

## Abbreviations

The following abbreviations are used in this manuscript:

DPPH	2,2-diphenyl-1-picrylhydrazyl
CUPRAC	Cupric Ion Reducing Antioxidant Capacity
ABTS	2,2-azinobis(3-ethylbenzothiazoline-6-sulfonic acid)
AChE	Acetylcholinesterase
BChE	Butrylcholinesterase
HPLC-DAD	High-Performance Liquid Chromatography-Diode Array Detector
CYP	Cytochrome
