# Supplementary material for: Integrated Phytochemical Profiling, In Vitro Bioactivity, Molecular Docking, and Pharmacokinetic–Toxicity Evaluation of Ficus carica Leaf Extracts
Source: Life (Basel). 2026 Sep 11;16(9):1518. doi: 10.3390/life16091518 (PMC13608220; doi:10.3390/life16091518)
Supplement: Supplementary file 1 [file life-16-01518-s001.zip › life-4507292-supplementary.pdf]

SUPPLEMENTARY MATERIAL

Integrated Phytochemical Profiling, *In Vitro* Bioactivity, Molecular Docking, and Pharmacokinetic–Toxicity Evaluation of *Ficus carica* Leaf Extracts

Bihter Şahin[1,2,*](https://orcid.org/0000-0001-8657-052X), Zafer Maşlakcı [1,2] (https://orcid.org/0000-0002-3661-8510), Gülşah Kani[1] (https://orcid.org/0009-0001-4516-7942), Özge Tokul Ölmez [3] (https://orcid.org/0000-0002-2093-4949)

[a]*Department of Chemistry and Chemical Processing Technologies, Susurluk Agriculture and Forest Vocational School, Bandırma Onyedi Eylül University, 10600, Balıkesir, Türkiye*

[b]*Department of Chemistry and Chemical Processing Technologies, Susurluk Agriculture and Forest Vocational School, Balıkesir University, 10600, Balıkesir, Türkiye*

[c]*Department of Chemistry, Faculty of Science, Muğla Sıtkı Koçman University, 48121, Muğla, Türkiye*

**Corresponding author: bihter.sahin@balikesir.edu.tr*

Abstract

Ficus carica L., a widely cultivated member of the Moraceae family, is recognized for its rich phytochemical composition and diverse biological activities; however, the influence of harvest season and extraction solvent on its bioactive profile remains insufficiently ex-plored. In this context, leaves collected from Dalaman (Muğla, Türkiye) in April and No-vember were comparatively evaluated in terms of phenolic profiles, antioxidant capacity, enzyme inhibitory potential, molecular docking interactions, and preliminary ADMET properties. Eight extracts were prepared using ethyl acetate, methanol, aqueous ethanol, and water, and phenolic composition was characterized by HPLC-DAD using 42 reference standards. Major contributors to bioactivity included hesperidin, rutin, chlorogenic acid, cynarin, naringenin, and 4-hydroxyresorcinol. Antioxidant activity was assessed via DPPH, ABTS, CUPRAC, and β-carotene–linoleic acid assays, while enzyme inhibitory ac-tivities against acetylcholinesterase, butyrylcholinesterase, and tyrosinase were deter-mined. Hesperidin reached its highest concentration in the water extract FC14 (80.06 mg/g). FC14 showed the strongest DPPH (IC_{50}: 54.46 ± 0.94 μg/mL), ABTS (IC_{50}: 10.75 ± 0.17 μg/mL), and CUPRAC ($A_{0.5}$: 95.38 ± 1.69 μg/mL) activities, whereas the ethyl acetate ex-tract FC11 showed the strongest β-carotene–linoleic acid activity (IC_{50}: 37.77 ± 2.11 μg/mL). The water extract FC24 showed the strongest AChE (IC_{50}: 135.52 ± 0.77 μg/mL) and tyrosi-nase (IC_{50}: 34.46 ± 1.05 μg/mL) inhibition. The results demonstrated that both harvesting season and extraction solvent significantly influenced the phytochemical composition and biological activities. Principal component analysis revealed clear discrimination among extracts, and molecular docking supported these findings by highlighting strong interac-tions of hesperidin and rutin with target enzymes. PCA explained 50.30% of the total var-iance. Molecular docking analysis revealed the strongest binding affinities for hesperidin toward AChE (−9.3 kcal/mol), hesperidin and rutin toward BChE (both −10.8 kcal/mol), and rutin toward tyrosinase (−10.3 kcal/mol). ADMET predictions suggested favorable oral drug-likeness for selected compounds, whereas others may require

further optimiza-tion. Overall, these findings indicate that *F. carica* leaves represent a promising source of natural phenolics with multifunctional bioactivity.

Keywords: *Ficus carica*, HPLC-DAD, antioxidant activity, enzyme inhibition activity, molecular docking, chemometric approach

Detailed Chemicals and Instrumentation

All chemicals and solvents used throughout the study were of analytical or HPLC grade. Methanol and acetic acid were obtained from Merck (Darmstadt, Germany). Ultrapure water was produced using a Milli-Q water purification system (Millipore, Bedford, MA, USA). Solid-phase extraction was performed using C_{18} cartridges (500 mg; Teknokroma, Barcelona, Spain; supplied by Finisterre Kimya, Türkiye). Reagents used for antioxidant activity assays, including β-carotene, linoleic acid, 2,2-diphenyl-1-picrylhydrazyl (DPPH), 2,2′-azino-bis(3-ethylbenzothiazoline-6-sulfonic acid) (ABTS), butylated hydroxyanisole (BHA) were purchased from Sigma–Aldrich (St. Louis, MO, USA). Copper (II) chloride, neocuproine, ammonium acetate, ferrozine, and ferrous chloride used in CUPRAC assays were also supplied by Sigma–Aldrich.

Acetylcholinesterase (AChE, electric eel), butyrylcholinesterase (BChE, horse serum), acetylthiocholine iodide, butyrylthiocholine chloride, 5,5′-dithiobis-(2-nitrobenzoic acid) (DTNB), and galantamine employed in anticholinesterase assays were obtained from Sigma–Aldrich. Mushroom tyrosinase, L-DOPA (3,4-dihydroxy-L-phenylalanine), and kojic acid used for tyrosinase inhibition assays, were purchased from the same supplier.

Phenolic and organic acid standards, including fumaric acid, gallic acid, protocatechuic acid, theobromine, theophylline, catechin, *p*-hydroxybenzoic acid, 6,7-dihydroxycoumarin, methyl-1,4-benzoquinone, vanillic acid, caffeic acid, vanillin, chlorogenic acid, *p*-coumaric acid, ferulic acid, cynarin, coumarin, propyl gallate, rutin, *trans*-2-hydroxycinnamic acid, ellagic acid, myricetin, fisetin, quercetin, *trans*-cinnamic acid, luteolin, rosmarinic acid, kaempferol, apigenin, chrysin, 1,4-dichlorobenzene, 4-hydroxyresorcinol, pyrocatechol, 4-hydroxybenzaldehyde, epicatechin, 2,4-dihydroxybenzaldehyde, hesperidin, oleuropein, naringenin, hesperetin, genistein, and curcumin were obtained from Sigma Aldrich (St. Louis, MO, USA).

Chromatographic studies were conducted using an HPLC system (Shimadzu Prominence-*i* series LC-2030C 3D Plus, Kyoto, Japan) equipped with a diode-array detector and an Inertsil ODS-3 column (5 µm, 4.6 × 250 mm) with a guard column (GL Sciences Inc., Tokyo, Japan). Prior to injection, samples were filtered through 0.45 µm membrane filters (Millipore, USA). Antioxidant and enzyme inhibition assays were performed using a microplate reader (BioTek Synergy H1 Microplate reader, BioTek (Santa Clara, CA, USA). Microscopic pollen analysis was carried out using an Olympus CX-31 light microscope (Olympus Corporation, Tokyo, Japan).

2.4. *Antioxidant and Enzyme İnhibitory Activity*

2.4.1. DPPH Radical Scavenging Activity

The DPPH radical scavenging potential of the essential oil and the ethyl acetate, methanol, ethanol with aqueous and water extracts of *F. carica* leaves was assessed following the procedure of Blois, with minor modifications. A 0.4 mM DPPH solution was obtained by

dissolving 16 mg of DPPH in 100 mL of methanol. Stock solutions of the extracts were first prepared, and subsequently, 40 µL of each extract, diluted in ethyl acetate, methanol, aqueous ethanol and water at varying concentrations, was added to 160 µL of freshly prepared DPPH solution in 96-well microplates. The mixtures were incubated at room temperature in the dark for 30 min, after which the absorbance was measured at 517 nm using a microplate spectrophotometer. For control measurements, 40 µL of the respective pure solvents (ethyl acetate, methanol, or water) replaced the extracts. The radical scavenging activity was then expressed by comparing the absorbance of the samples with that of the controls [37].

2.4.2. ABTS Cation Radical Scavenging Activity

The ABTS radical cation scavenging activity of the *F. carica* leaves extracts was evaluated according to the method described by Re. In this assay, 40 µL of the sample stock solutions prepared at different concentrations were mixed with 160 µL of ABTS radical solution in a 96-well microplate. After the mixtures were incubated for a specified time at room temperature, the absorbance was recorded at 734 nm using a microplate spectrophotometer. BHA (butylated hydroxyanisole) was used as a positive control for all comparison of antioxidant capacity. The radical scavenging activity was calculated based on the decrease in absorbance compared to the control [38].

$$ABTS\ inhibition\ activity\ (inhibition\%) = A_{Control} - A_{Sample} / A_{Control}\ x\ 100 \quad (S1)$$

2.4.3. CUPRAC Activity Method

The cupric ion (Cu^{2+}) reducing antioxidant capacity of the *F. carica* leaves extracts was determined using the CUPRAC method as described by Apak et al., with slight modifications. Briefly, 40 µL of each sample solution at various concentrations was mixed with 60 µL of ammonium acetate buffer (pH 7.0) and 100 µL of the neocuproine–Cu(II) reagent in a 96-well microplate. The reaction mixtures were incubated at 25°C for 1 hour. After incubation, the absorbance was measured at 450 nm using a microplate reader.

The antioxidant activity was expressed in terms of the concentration required to achieve an absorbance value of 0.5 ($A_{0.5}$). BHA (butylated hydroxyanisole) was used as a positive control for comparison of antioxidant capacity [39].

2.4.4. β-carotene-linoleic Acid Decolorization Activity

The antioxidant activity of the samples was also evaluated using the *β*-carotene–linoleic acid bleaching method, as described by Miller, with slight modifications. For the assay, 40 µL of the sample stock solutions at different concentrations were mixed with 160 µL of *β*-carotene solution, forming an emulsion. The emulsified mixtures were transferred to 96-well microplates, and the initial absorbance was recorded at 490 nm. Following the initial measurement, the microplates were incubated at 45°C, and absorbance measurements were repeated every 30 minutes for approximately 120 minutes, until the *β*-carotene in the control sample was fully discolored [40].

2.4.5. Anticholinesterase Activity

The inhibitory activity against acetylcholinesterase and butyrylcholinesterase were assessed using a modified spectrophotometric procedure based on the classical method of

Ellman [41]. In this assay, acetylthiocholine iodide and butyrylthiocholine iodide served as the enzymatic substrate, while DTNB [5,5'-dithiobis-(2-nitrobenzoic acid)] acted as the chromogenic reagent for the detection of cholinesterase inhibition. In brief, the reaction mixture consisted of 150 μL of 100 mM sodium phosphate buffer (pH 8.0), 10 μL of the test sample (prepared in ethanol at various concentrations), and 20 μL of AChE solution (5.32×10^{-3} U), followed by incubation for 15 minutes at 25 °C. Subsequently, 10 μL of 0.5 mM DTNB was introduced. The enzymatic reaction was initiated by the addition of 10 μL of 0.71 mM acetylthiocholine iodide and butyrylthiocholine iodide.

The generation of the yellow-colored 5-thio-2-nitrobenzoate ion, formed by the reaction between DTNB and thiocholine (a product of AChE and BChE-mediated hydrolysis of the substrate), was monitored at 412 nm using a 96-well multimode plate reader (BioTech, USA). Galantamine was used as the positive control.

The percentage inhibition of AChE and BChE activity was determined using the equation:

$$\text{Inhibition (\%)} = \left(\frac{\text{Activity}_0 - \text{Activity}_t}{\text{Activity}_0}\right) \times 100 \quad \text{(S2)}$$

where $Activity_0$ denotes the enzyme activity in the absence of inhibitor, and $Activity_t$ represents the activity in the presence of the test sample.

2.4.6. Tyrosinase Activity

The tyrosinase inhibition activity of *F. carica* leaf extracts was evaluated spectrophotometrically according to the method of Hearing [42]. Mushroom tyrosinase was used as the enzyme and L-DOPA as the substrate. In a 96-well microplate, 150 μL of 0.005 M phosphate buffer (pH 6.8), 10 μL of extract solutions at various concentrations, and 20 μL of mushroom tyrosinase were added sequentially. The mixtures were stirred for 3 min and incubated at 37 °C for 10 min. Following incubation, 20 μL of L-DOPA solution was added, and the kinetic absorbance was immediately recorded at 475 nm for 10 min at 37 °C. The results are presented in Table 2.

Table S1. Gradient elution program used for HPLC analysis.

Time	Mobile Phase B<br>100% methanol	Mobile Phase A<br>0.1% acetic acid in water
0-6 (6 min)	2%	98%
7-11 (4 min)	5%	95%
12	6%	94%
13-24 (9 min)	10%	90%
25-31 (6 min)	25%	75%
32-36 (4 min)	30%	70%
37-42 (5 min)	40%	60%
43-47 (4 min)	42%	58%
48-52 (4 min)	54%	46%
53-62 (9 min)	55%	45%
63-66 (3 min)	56%	44%

67-71 (4 min)	65%	35%
72-74 (2 min)	100%	0%
75-76 (2 min)	50%	50%
77	2%	98%

Table S2. Retention time, calibration curves, regression coefficient (R^2), linearity ranges, LODs, and recoveries of phenolic standards at 254 nm.

No	Compounds	Calibration equation	R^{2b}	RT [a] (min)	Linear range (mg/mL)	LOD [c] (µg/mL)	RSD%	Recovery
1	**Fumaric acid**	1988,9x - 46	0.9998	14.014	5.08-162.5	1	1.35	101.36
2	**Gallic acid**	45540x - 84	0.995	15.225	1.95-62.5	2	7.41	108.00
3	**Protocatechic acid**	65753x - 69	0.9999	24.683	0.57-290	0.1	0.22	100.22
4	**Theobromine**	3942,7x + 81	0.9983	26.077	2.35-2407	1	-6.37	94.02
5	**Theophlline**	36694x + 68	0.9998	29.449	0.88-450	0.25	-3.22	96.88
6	**Catechin**	2611,2x + 74	0.9972	30.238	3.8-1950	1	-4.01	96.14
7	**4-hydroxy benzoic acid**	123758x + 7	0.9997	31.691	0.16-162.5	0.05	-6.66	93.75
8	**6,7-dihydroxycoumarin**	27607x + 20	0,9963	33,435	0,93-475	0,1	-1,88	98,16
9	**Methyl-1,4-benzoquinon**	37060x - 637	0,9934	33,999	6,05-387,5	1	3,37	103,37
10	**Vanilic acid**	66764x + 46	0,9998	34,688	0,39-200	0,1	0,16	100,16
11	**Caffeic acid**	49533x + 21	0,9957	35,198	0,46-235	0,1	3,83	103,83
12	**Vanillin**	21426x + 19	0,9995	37,12	4,56-1167,5	1	-4,07	95,93
13	**Chlorogenic acid**	46920x - 36	0,9995	38,88	1,81-116,09	1	-1,38	98,62
14	***p*-Coumaric acid**	17265x + 34	0,9951	40,819	1,25-1277,5	0,5	-3,02	96,98
15	**Ferulic acid**	42245x + 11	0,9992	42,921	0,8-410	0,1	-4,81	95,19
16	**Cynarin**	42559x - 2E	0.9959	43.853	32-512	20	-5.09	94.91
17	**Coumarin**	81802x + 15	0.9968	44.913	0.71-189	0.1	-3.70	96.30
18	**Prophylgallate**	29731x - 12	1	46.984	1.86-952.5	0.5	-4.56	95.44
19	**Rutin**	47899x + 56	0.9997	47.527	0.46-235	0.1	-0.72	99.28
20	***Trans*-2-hydroxycinnamic acid**	53442x + 10	0.9996	48.072	0.35-355	0.1	-5.29	94.71
21	**Ellagic acid**	235073-7E	0.9808	50.005	15-250	5	-4.51	95.49
22	**Myricetin**	136859x + 7	0.995	50.368	0.35-22.5	0.1	-2.59	97.41
23	**Fisetin**	100784x + 1	0.9984	51.243	0.28-72.5	0.1	-3.16	96.84
24	**Quercetin**	89397x - 80	0.9997	55.424	0.2-200	0.05	4.35	104.35
25	***Trans* cinnamic acid**	88190x + 15	0.9997	55.92	0.78-375	0.1	-4.43	95.57
26	**Luteolin**	89569x - 62	0.9995	57.872	0.59-150	0.1	-0.18	99.82
27	**Rosmarinic acid**	100183x + 5	0.9995	60.813	0.36-185	0.1	-1.78	98.22
28	**Kaemferol**	92669x + 58	0.9995	62.485	0.39-200	0.1	-2.27	97.73
29	**Apigenin**	71990x + 62	0.9996	64.071	0.66-170	0.2	-1.20	98.80
30	**Chyrsin**	26957x + 28	0.9994	72.779	0.24-250	0.1	-3.20	96.80
31	**4-hydroxylresorkinol**	389,3x + 10	0.9991	73.061	3.78-1937.5	1	-2.56	97.44
32	**1.4-dichlorobenzene**	482,7x + 10	0.9996	73.813	7.1-1817.5	1	4.18	104.18
1	**Pyrocatechol**	3772,8x + 23	0.9925	24.658	1.39-178	0.5	-4.09	95.91
2	**4-hydroxy benzaldehid**	34376x + 42	0.9996	33.367	0.08-10.5	0.01	0.67	100.67
3	**Epicatechin**	2097,6x + 79	0.998	35.278	1.43-183.5	0.1	-4.11	95.89
4	**2.4-dihydroxybenzoic acid**	41139x + 16	0.9998	38.616	0.41-52	0.1	-1.08	98.92
5	**Hesperedin**	2949,5x + 90	0.9995	47.381	1.92-246	0.5	-1.87	98.13
6	**Oleuropein**	6962,8x + 42	0.9995	49.548	0.89-57	0.1	-1.85	98.15
7	**Naringenin**	5906,1x + 8	0.9992	55.518	1-130	0.1	-2.66	97.34
8	**Hesperetin**	4524,4x + 10	0.9994	57.47	0.94-120	0.1	-0.91	99.09
9	**Genistein**	69160x + 57	0.9996	57.739	0.1-6.5	0.01	-2.60	97.40
10	**Curcumin**	15850x + 14	0.9932	72.895	2-32.5	0.5	2.42	102.42

[a] RT: Retention time of the compound in minutes, [b] R^2: linearity of the calibration graph, [c] LOD: Limit of Detection in µg/mL, [d] RSD: Percentage relative standard deviation.

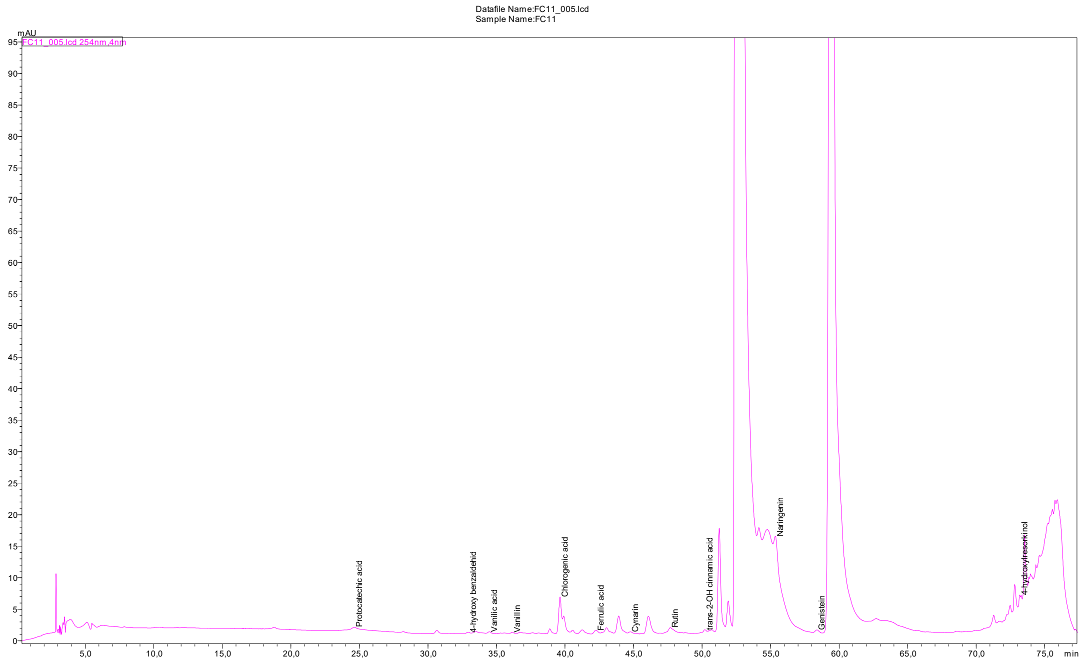

Figure S1. HPLC-DAD chromatogram of the FC11 extract.

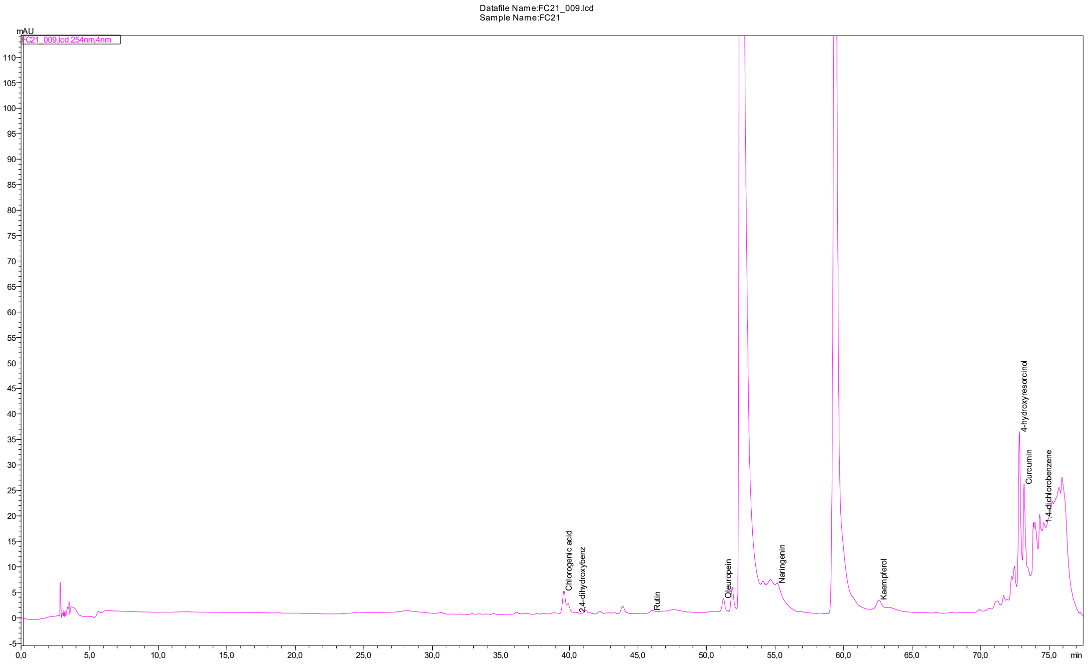

Figure S2. HPLC-DAD chromatogram of the FC21 extract.

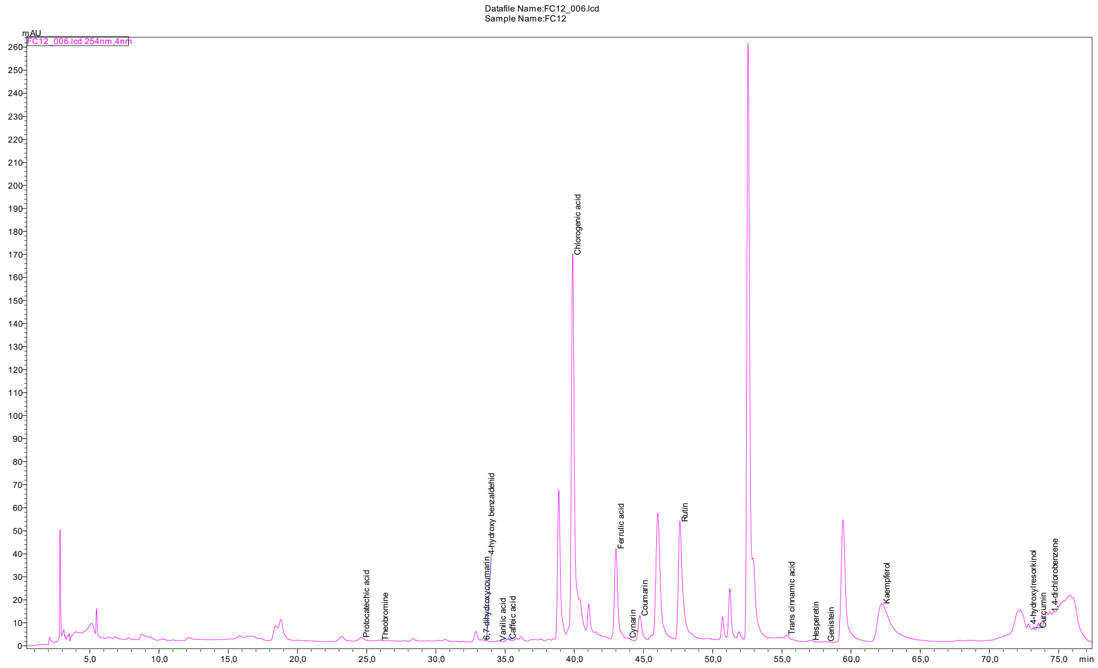

Figure S3. HPLC-DAD chromatogram of the FC12 extract.

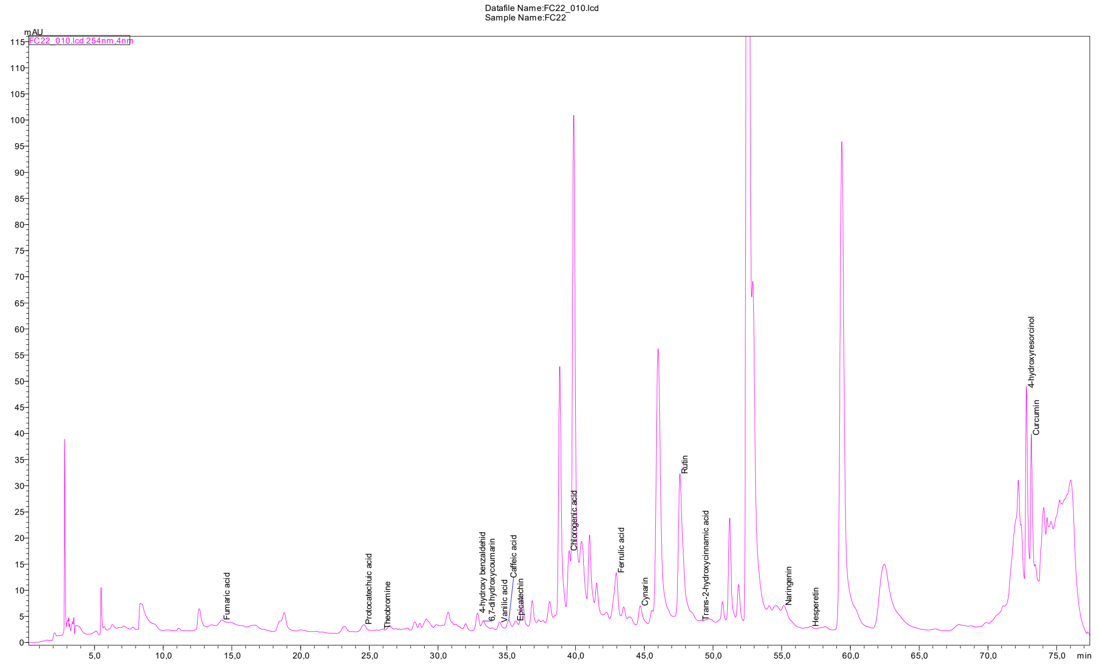

Figure S4. HPLC-DAD chromatogram of the FC22 extract.

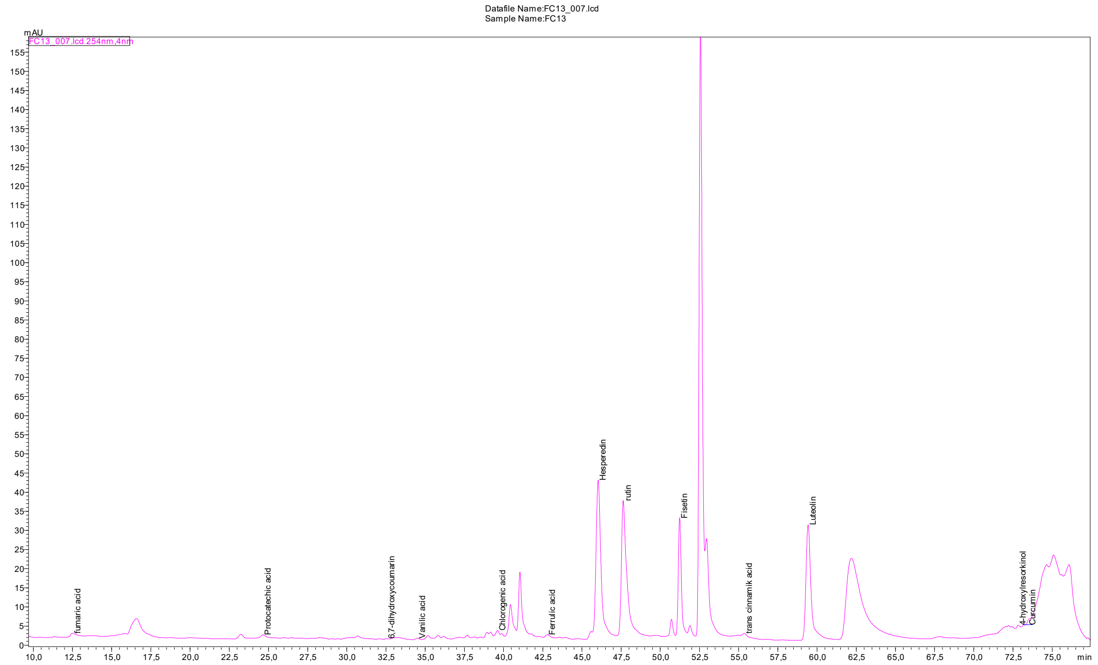

Figure S5. HPLC-DAD chromatogram of the FC13 extract.

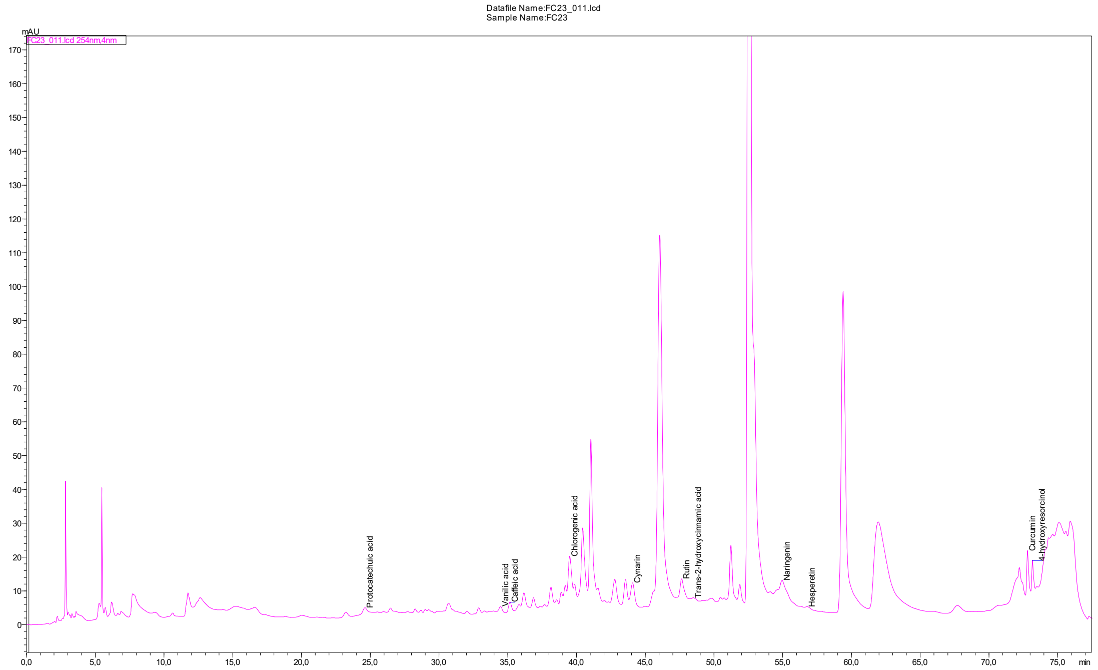

Figure S6. HPLC-DAD chromatogram of the FC23 extract.

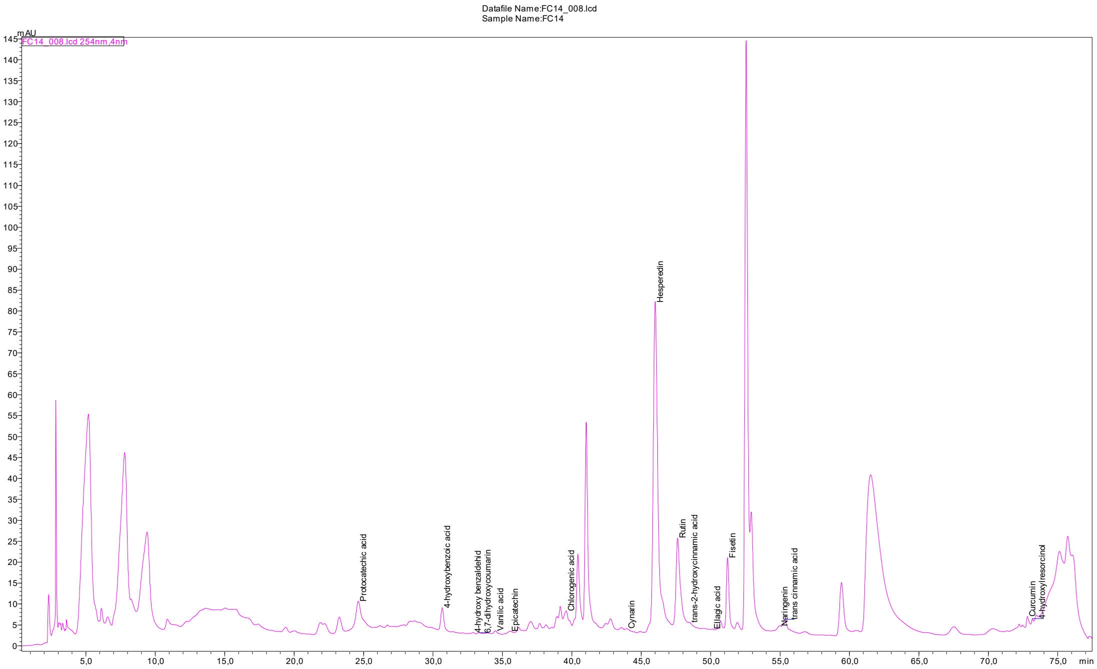

Figure S7. HPLC-DAD chromatogram of the FC14 extract.

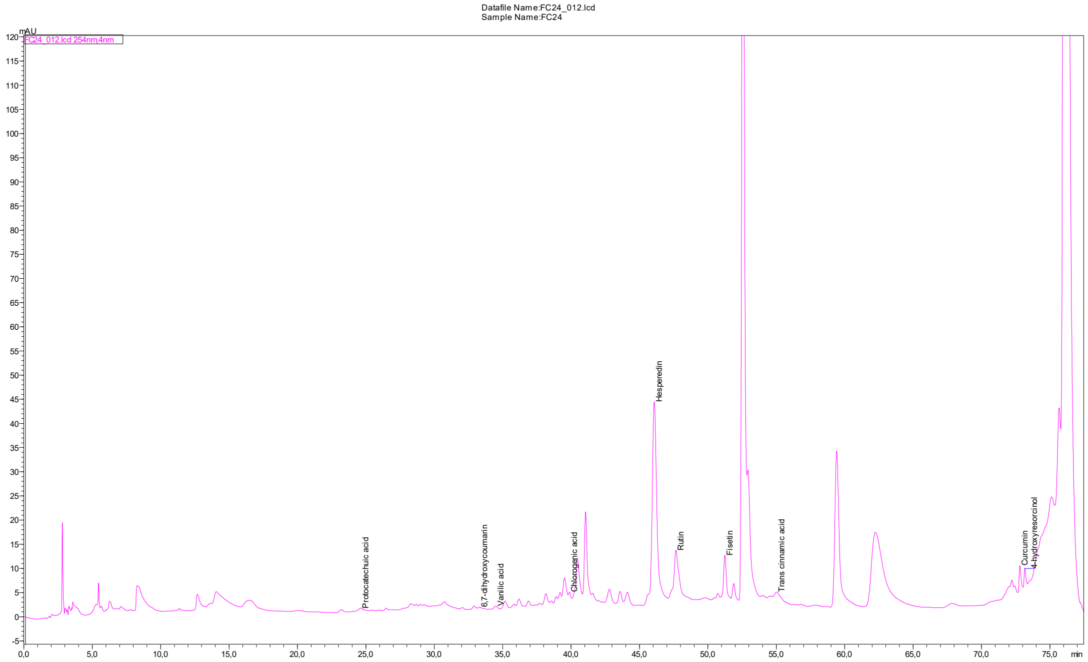

Figure S8. HPLC-DAD chromatogram of the FC24 extract.

Table S3. Loading, Eigenvalue, Variance (%), and Cumulative Variance (%) Values of Different Solvent Extracts of *F. carica* Leaves Harvested in April and November.

VARIABLES	PC1	PC2	PC3	PC4	PC5	PC6	PC7
**Fumaric acid**	-0.099	0.063	-0.059	-0.090	-0.386	0.337	-0.032
**Protocatechic acid**	-0.189	0.145	-0.020	-0.116	0.294	-0.060	-0.236
**Theobromine**	**0.232**	0.204	0.077	-0.168	-0.091	-0.024	-0.091
**4-hydroxy benzaldehid**	-0.006	0.082	**0.233**	-0.201	-0.230	0.022	-0.340
**Vanillin**	0.047	-0.113	0.076	0.337	-0.021	-0.003	-0.441
**Vanilic acid**	-0.072	**0.269**	0.125	0.234	-0.105	-0.035	0.002
**6,7-Dihydroxycoumarin**	-0.061	**0.269**	-0.094	-0.138	-0.183	-0.252	-0.011
**Chlorogenic acid**	**0.242**	0.205	-0.130	0.029	0.104	0.029	0.058
**2,4-Dihydroxybenzoic acid**	0.072	-0.269	-0.125	-0.234	0.105	0.035	-0.002
**Ferulic acid**	**0.258**	**0.216**	-0.093	-0.022	0.029	0.006	-0.007
**Caffeic acid**	0.045	**0.247**	0.166	-0.142	0.222	0.130	0.033
**Epicatechin**	-0.004	0.107	**0.242**	-0.283	-0.119	-0.074	-0.255
**Cynarin**	0.076	0.175	**0.235**	0.075	0.212	0.143	-0.245
**Coumarin**	**0.241**	0.197	-0.155	0.035	0.088	0.008	0.043
**Hesperidin**	-0.279	0.114	-0.128	-0.034	0.056	-0.225	-0.079
**Rutin**	0.086	**0.284**	-0.071	-0.154	-0.188	0.076	-0.068
**Trans-2-hydroxycinnamic acid**	0.015	-0.054	**0.328**	0.178	-0.104	-0.200	0.138
**Trans cinnamic acid**	-0.054	0.038	-0.355	-0.124	0.030	-0.201	0.098
**Naringenin**	0.010	-0.109	**0.255**	0.171	0.171	0.327	0.162
**Hesperetin**	0.187	0.174	0.197	-0.083	0.044	0.209	0.233
**Genistein**	**0.215**	**0.212**	-0.163	-0.046	0.088	0.009	0.144
**Kaempferol**	**0.244**	0.191	-0.159	0.029	0.091	0.009	0.043
**4-Hydroxylresorcinol**	0.110	-0.111	0.174	-0.362	-0.075	0.050	-0.048
**Curcumin**	0.095	-0.146	0.168	-0.356	-0.037	0.092	0.006
**Oleuropein**	0.072	-0.269	-0.125	-0.234	0.105	0.035	-0.002
**Fisetin**	-0.246	0.087	-0.196	-0.003	-0.183	0.136	-0.016
**Ellagic acid**	-0.205	0.119	-0.038	-0.094	0.272	-0.109	-0.290
**Luteolin**	-0.131	0.034	-0.178	0.023	-0.306	0.368	0.041
**DPPH˙ assay**	-0.270	0.111	-0.079	-0.011	0.061	0.344	0.014
**ABTS˙+ assay**	-0.287	0.073	0.037	-0.162	0.036	-0.040	0.255
**β-Carotene-linoleic acid**	-0.053	**0.236**	0.131	0.275	-0.150	-0.049	0.022
**CUPRAC**	-0.271	0.127	-0.061	-0.048	0.175	0.179	-0.112
**Acetylcholinesterase activity**	0.163	-0.092	-0.101	0.047	-0.343	-0.253	-0.115
**Butyrylcholinesterase activity**	0.159	-0.090	-0.255	0.106	-0.084	0.266	-0.232
**Tyrosinase activity**	-0.187	0.103	0.200	-0.096	-0.142	-0.131	0.342
**Eigenvalue**	7.472	5.083	3.957	3.651	2.430	1.384	1.023
**Proportion**	30.0	20.3	15.8	14.6	9.7	5.5	4.1
**Cumulative**	30.0	50.3	66.0	80.6	90.4	95.6	1.0

Table S4. The Score Values of Different Solvent Extracts of *F. carica* Leaves Harvested in April and November

SAMPLES	PC1	PC2	PC3	PC4	PC5	PC6	PC7
**FC11**	1.005	-2.212	1.194	3.646	-0.216	-0.015	-1.936
**FC21**	1.537	-5.247	-1.969	-2.529	1.070	0.160	-0.009
**FC12**	**5.134**	**3.853**	-2.438	0.380	0.900	0.039	0.189
**FC22**	1.326	1.352	**4.032**	-2.759	-2.123	-0.179	-0.714
**FC13**	-2.787	0.655	-2.803	0.247	-3.134	1.679	0.180
**FC23**	-0.634	-0.241	2.952	1.071	1.883	1.540	2.018
**FC14**	-4.379	2.330	-0.596	-1.019	2.779	-0.499	-1.273
**FC24**	-1.203	-0.490	-0.373	0.963	-1.161	-2.723	1.545

Table S5. Predicted Physicochemical and Drug-Likeness Properties of the Selected Bioactive Phenolic Compounds from *F. carica* Leaf Extracts.

**Compound**	**MW(g /mol)**	**HB A**	**HB D**	**TPSA (Å²)**	**Rotatable bonds**	**Consensus LogP**	**ESOL LogS**	**GI absorption**	**BBB permeant**	**P-gp substrate**	**Lipinski violations**	**Bioavailability score**
Hesperidin	610.56	15	8	234.29	7	-1.55	-3.28	Low	No	Yes	3	0.17
Rutin	610.52	16	10	269.43	6	-2.74	-2.33	Low	No	No	3	0.17
Chlorogenic acid	354.31	9	6	164.75	5	-0.71	-1.62	Low	No	No	1	0.11
Cynarin	516.45	12	7	211.28	9	0.67	-3.65	Low	No	Yes	3	0.11
Ellagic acid	302.19	8	4	141.34	0	1.12	-4.36	High	No	No	0	0.55
Kaempferol	286.24	6	4	111.13	1	1.20	-3.33	High	No	No	0	0.55
Naringenin	272.25	5	3	86.99	1	1.87	-3.49	High	No	Yes	0	0.55
Ferulic acid	194.18	4	2	66.76	3	1.29	-2.11	High	Yes	No	0	0.85

Table S6. Predicted pharmacokinetic and toxicity-related properties of the selected bioactive phenolic compounds identified in *F. carica* leaf extracts using pkCSM.

**Compound**	**Water solubility**(log mol/L)	**Caco-2 permeability**(log Papp in 10^{-6} cm/s)	**Intestinal absorption** (%)	**VDss** (log L/kg)	**BBB permeability** (log BB)	**CYP inhibition**	**Total clearance**	**AMES toxicity**	**Hepatotoxicity**	**Skin sensitization**
Hesperidin	-3.014	0.505	31.481	0.996	-1.715	CYP2D6, CYP3A4, CYP1A2, CYP2C19, CYP2C9, CYP2D6, CYP3A4; No	0.211	No	No	No
Rutin	-2.892	-0.949	23.446	1.663	-1.899	CYP2D6, CYP3A4, CYP1A2, CYP2C19, CYP2C9, CYP2D6, CYP3A4; No	-0.369	No	No	No
Chlorogenic acid	-2.449	-0.84	36.377	0.581	-1.407	CYP2D6, CYP3A4, CYP1A2, CYP2C19, CYP2C9, CYP2D6, CYP3A4; No	0.307	No	No	No
Cynarin	-2.962	-1.092	30.305	1.849	-1.983	CYP2D6; No, CYP3A4; Yes, CYP1A2, CYP2C19, CYP2C9, CYP2D6, CYP3A4; No	-0.056	No	No	No
Ellagic acid	-3.181	0.335	86.684	0.375	-1.272	CYP2D6; No, CYP3A4; No, CYP1A2; Yes, CYP2C19, CYP2C9, CYP2D6, CYP3A4; No	0.537	No	No	No
Kaempferol	-3.04	0.032	74.29	1.274	-0.939	CYP2D6; No, CYP3A4; No, CYP1A2; Yes, CYP2C19, CYP2C9, CYP2D6, CYP3A4; No	0.477	No	No	No
Naringenin	-3.224	1.029	91.31	-0.015	-0.578	CYP2D6; No, CYP3A4; No, CYP1A2; Yes, CYP2C19, CYP2C9, CYP2D6, CYP3A4; No	0.06	No	No	No
Ferulic acid	-2.817	0.176	93.685	-1.367	-0.239	CYP2D6, CYP3A4, CYP1A2,	0.623	No	No	No

Compound	Water solubility(log mol/L)	Caco-2 permeability(log Papp in 10^{-6} cm/s)	Intestinal absorption (%)	VDss (log L/kg)	BBB permeability (log BB)	CYP inhibition	Total clearance	AMES toxicity	Hepatotoxicity	Skin sensitization
						CYP2C19, CYP2C9, CYP2D6, CYP3A4; No				

Table S7. Predicted Toxicity Profiles of Selected Bioactive Phenolic Compounds from *F. carica* Leaf Extracts Using ProTox-II.

Compound	Predicted LD50 (mg/kg)	Toxicity class	Hepatotoxicity	Carcinogenicity	Immunotoxicity	Mutagenicity	Cytotoxicity
Hesperidin	12000	6	0.81	0.93	0.99	0.90	0.52
Rutin	5000	5	0.80	0.91	0.98	0.88	0.64
Chlorogenic acid	5000	5	0.72	0.68	0.99	0.93	0.80
Cynarin	5000	5	0.71	0.65	0.98	0.85	0.84
Ellagic acid	2991	4	0.83	0.59	0.81	0.84	0.90
Kaempferol	3919	5	0.68	0.72	0.96	0.52	0.98
Naringenin	2000	4	0.67	0.62	0.88	0.83	0.59
Ferulic acid	1772	4	0.51	0.61	0.91	0.96	0.88

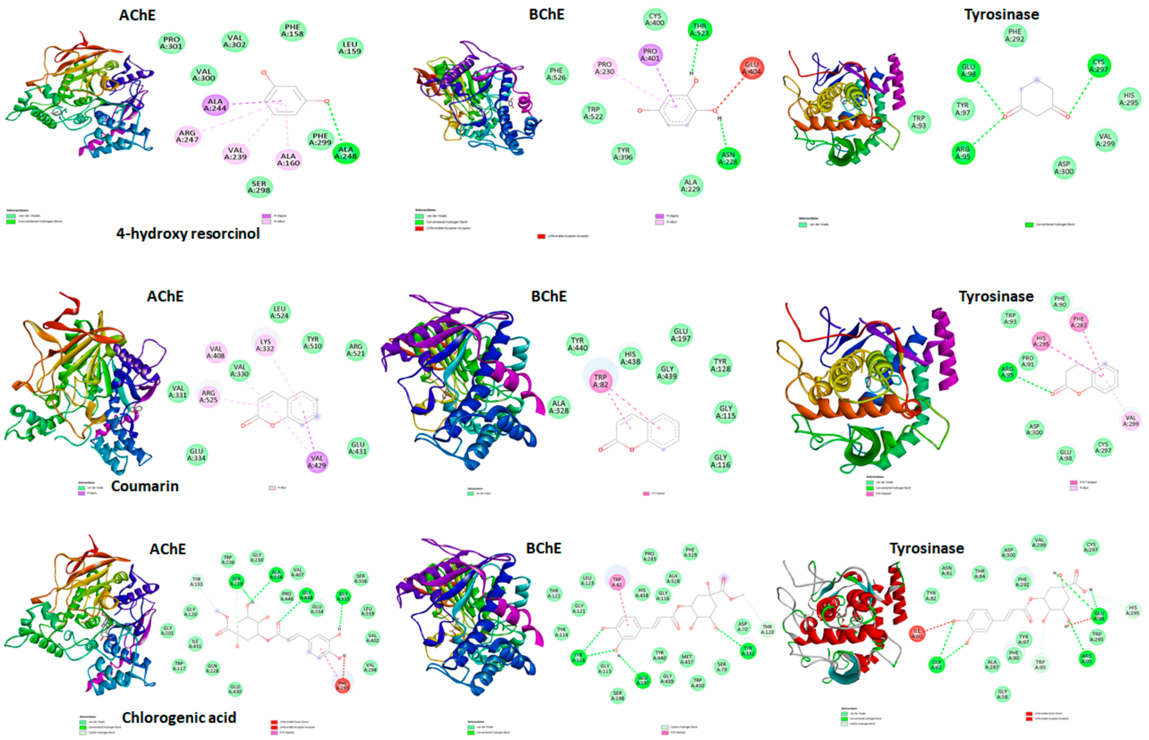

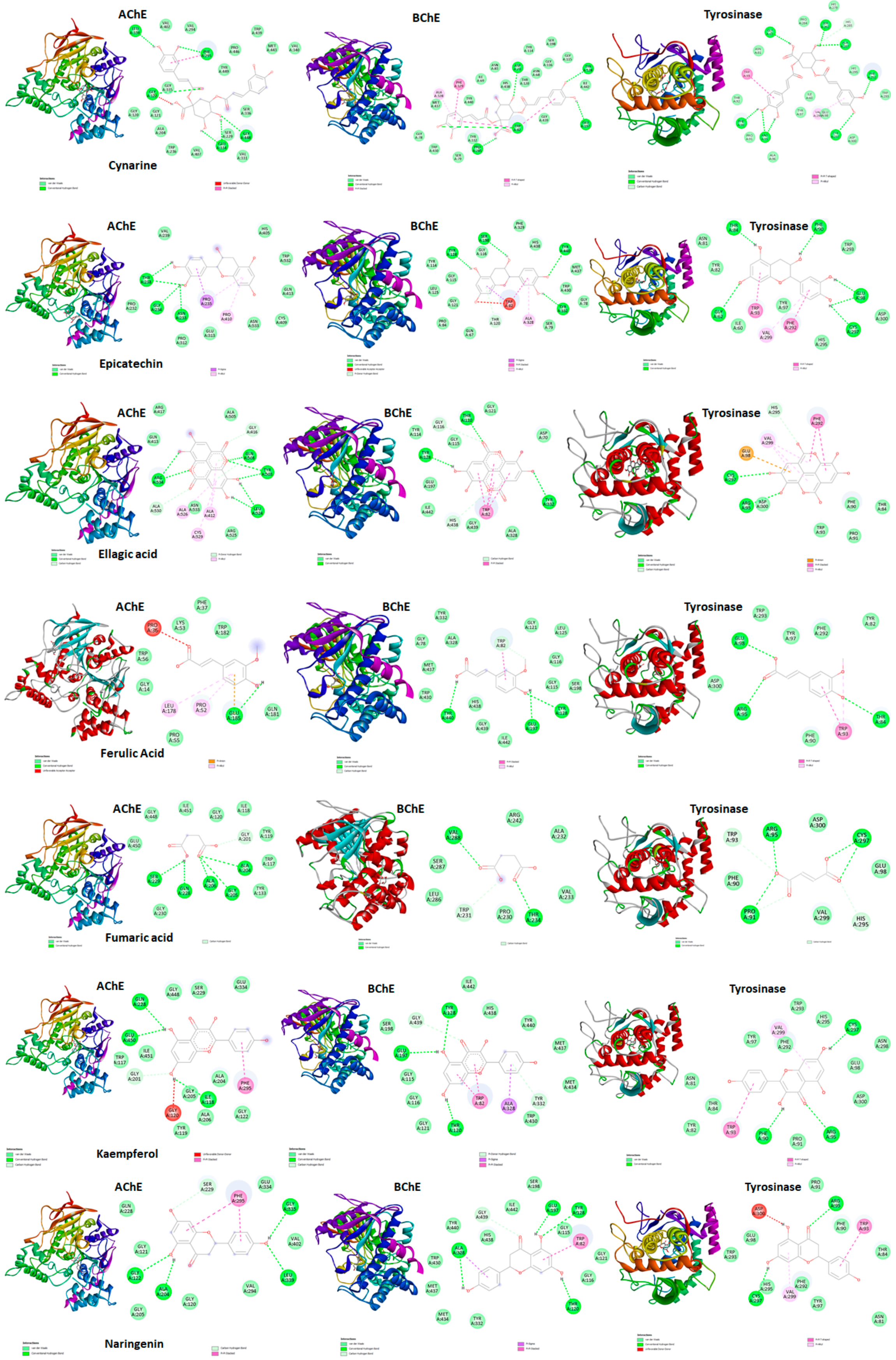

AChE
BChE
Tyrosinase
Cynarine
AChE
BChE
Tyrosinase
Epicatechin
AChE
BChE
Tyrosinase
Ellagic acid
AChE
BChE
Tyrosinase
Ferulic Acid
AChE
BChE
Tyrosinase
Fumaric acid
AChE
BChE
Tyrosinase
Kaempferol
AChE
BChE
Tyrosinase
Naringenin

Figure S9. Docking results of ligands in catalytic pocket of enzymes for compounds. AChE, acetylcholinesterase; BChE, butyrylcholinesterase.
